# A Comparison of Four Methods for the Analysis of N-of-1 Trials

**DOI:** 10.1371/journal.pone.0087752

**Published:** 2014-02-04

**Authors:** Xinlin Chen, Pingyan Chen

**Affiliations:** Department of Biostatistics, School of Public Health, Southern Medical University, Guangzhou, Guangdong province, China; Department of Preventive Medicine and Biostatistics, College of fundamental Medicine, Guangzhou University of Chinese Medicine, Guangzhou, Guangdong province, China; University of Rochester, United States of America

## Abstract

**Objective:**

To provide a practical guidance for the analysis of N-of-1 trials by comparing four commonly used models.

**Methods:**

The four models, paired t-test, mixed effects model of difference, mixed effects model and meta-analysis of summary data were compared using a simulation study. The assumed 3-cycles and 4-cycles N-of-1 trials were set with sample sizes of 1, 3, 5, 10, 20 and 30 respectively under normally distributed assumption. The data were generated based on variance-covariance matrix under the assumption of (i) compound symmetry structure or first-order autoregressive structure, and (ii) no carryover effect or 20% carryover effect. Type I error, power, bias (mean error), and mean square error (MSE) of effect differences between two groups were used to evaluate the performance of the four models.

**Results:**

The results from the 3-cycles and 4-cycles N-of-1 trials were comparable with respect to type I error, power, bias and MSE. Paired t-test yielded type I error near to the nominal level, higher power, comparable bias and small MSE, whether there was carryover effect or not. Compared with paired t-test, mixed effects model produced similar size of type I error, smaller bias, but lower power and bigger MSE. Mixed effects model of difference and meta-analysis of summary data yielded type I error far from the nominal level, low power, and large bias and MSE irrespective of the presence or absence of carryover effect.

**Conclusion:**

We recommended paired t-test to be used for normally distributed data of N-of-1 trials because of its optimal statistical performance. In the presence of carryover effects, mixed effects model could be used as an alternative.

## Introduction

**Figure 1:**
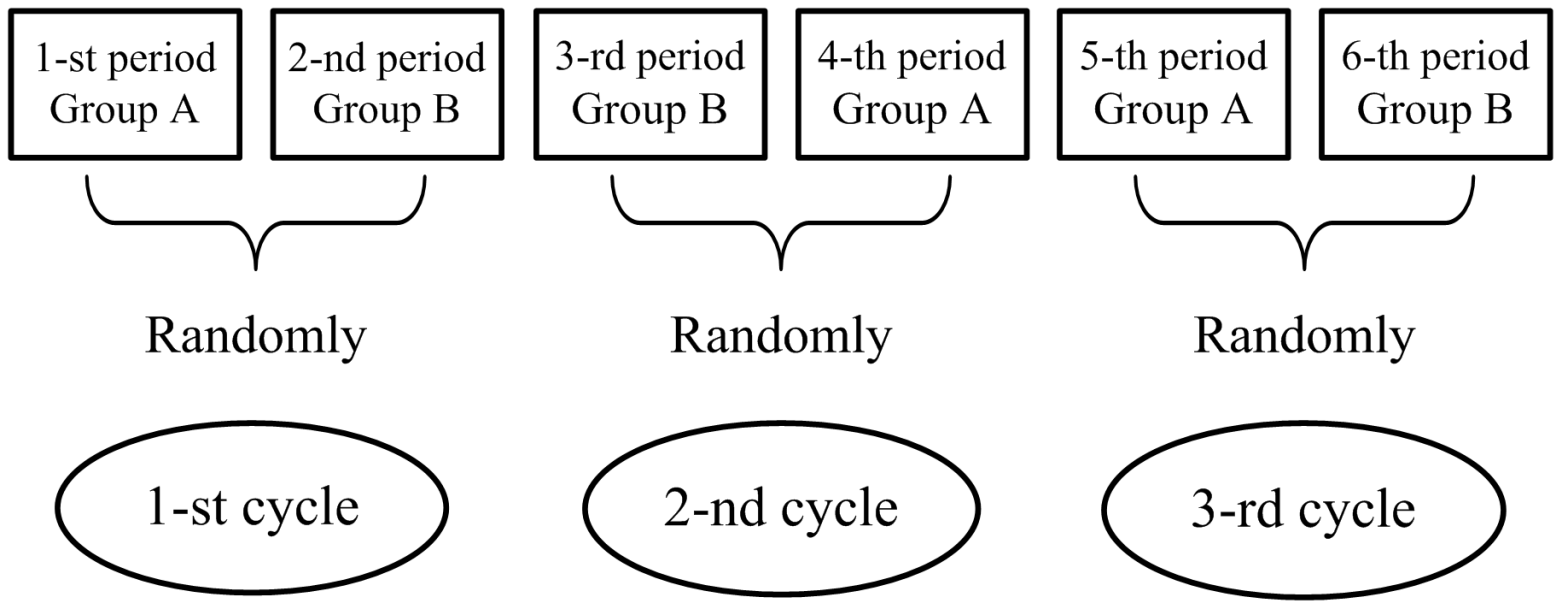
3-cycles N-of-1 trials. (There is a washout period between successive treatment periods).

N-of-1 trials (single case of randomized controlled trials, randomized controlled trial in individual patient) are multicycle, double-blinded controlled cross-over trials based on individuals [1], [2], [3], [4]. N-of-1 trials are designed to test the effect difference of two treatments which are conventionally labeled as Group A (test group) and Group B (control group). The two periods in each cycle are randomly assigned to different treatments for each subject with a washout period. Figure 1 shows a typical 3-cycles N-of-1 trials.

Since N-of-1 trials were first introduced in an experimental paradigm in 1945 [5], they have been increasingly utilized in social, educational sciences, biomedical, clinical areas [6], and notably in medical area including rheumatism [7], pediatric rheumatism [8], arthritis pain [9], chronic neuropathic pain [10], insomnia [11], heart disease [12], chronic obstructive pulmonary disease [13], and pediatric oncology [14]. It was reported that 50 of 57 N-of-1 trials provided a significantly clinical effect, while 26.3% of the trials (15 trials) consequently led the physician to alter the patients’ treatment [15].

Evidence-Based Medicine Working Group suggested that N-of-1 trials provided the strongest evidence for the decisions of the individual patient [16]. However, N-of-1 trials have not been widely used. One reason was that N-of-1 trials required relatively stable symptoms or diseases, medications with short half-lives, and rapid measurable responses [3], [17]. Another important reason was related to the difficulties about the statistical analysis of the data [18].

To analyze the normally distributed data of N-of-1 trials, a number of methods have been proposed. Gabler et al reported that 52% of 108 articles used a visual/graphical representations without statistical comparison, 44% t-test, and 24% pooled analysis [19]. Visual analysis which plotted the data on a graph was commonly used [20], though its validity remained unclear. The parametric tests, such as z-test, two samples t-test, paired t-test and analysis of variance were widely applied to analyze such data [21]. Meta-analysis of summary data (short for meta-analysis) was proposed to estimate the pooled treatment effect for more than two subjects [22]. Individual participant data meta-analysis used linear mixed models to estimate treatment effect while accounting for correlations deriving from the individuals [8], [22], [23], [24]. Mixed effects model and generalized estimating equation based on the effect difference of two treatments in each cycle were established [18], [25].

However, it remained unclear which method should be adopted to provide more robust inferences for data of N-of-1 trials. To provide a practical guidance for the analysis of N-of-1 trials, we conducted a simulation study of 3-cycles and 4-cycles N-of-1 trials to compare the performance of four methods under various variance-covariance structures.

## Methods

For simplicity, the four methods are introduced below in 3-cycles design.

### Paired t-test (Model 1)

In N-of-1 trials, each cycle with two periods which are assigned to Group A or Group B is considered as a pair. For example, in 3-cycles N-of-1 trials with 10 subjects enrolled, 30 pairs of data are delivered because each subject provides 3 pairs of data. Paired t-test (Model 1) is then used to analyze the 30 pairs of data altogether, which does not account for between-subject effects.

### Mixed Effects Model of Difference (Model 2)

The difference of the two groups in the same cycle is calculated. There are three differences for each subject. Mixed effects model of difference can be formulated as
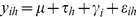
(1)where *y_ih_* denotes the *i*-th (*i* = 1, 2, …, *n*) subject’s difference for the *h*-th (*h* = 1, 2, 3) cycle. The intercept *μ* represents the overall mean of effect difference of Group A and Group B. *τ_h_* represents cycle effect for the *h*-th cycle. *γ_i_* indicates random effects of the *i*-th subject. *ε_ih_* represents the *i*-th subject’s random error for the *h*-th cycle.

If there is only one subject (*n* = 1), Model 2 reduces to: 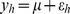. *y_h_* denotes the subject’s difference for the *h*-th (*h* = 1, 2, 3) cycle. *ε_h_* represents random error for the *h*-th cycle. So the model is identical to paired t-test (Model 1) when *n* = 1.

### Mixed Effects Model (Model 3)

We assume that carryover effect is only caused by treatment effect of the previous period and not by other periods. All the periods except the first period have carryover effect. Therefore, there are three kinds of effects: no carryover effect, carryover effect of Group A (*λ_A_*) and carryover effect of Group B (*λ_B_*). *λ_A_* and *λ_B_* represent carryover effect left over by Group A and Group B in the previous period respectively. Based on mixed effects model established by Zucker et al [25], we add period effect and carryover effect into the model. Considering all response values in six periods, mixed effects model (Model 3) is set as
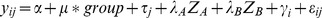
(2)where *y_ij_* denotes the *i*-th(*i* = 1, 2, …, *n*) subject’s response value for the *j*-th (*j* = 1, 2, …6) period. *α* is the intercept of the model. *μ* means the overall mean of effect difference between two groups. *group* = 1 if the subject is assigned to Group A, and *group* = 0 if the subject is assigned to Group B. *τ_j_* represents period effect for the *j*-th period. *λ_A_* and *λ_B_* represent carryover effect of Group A and Group B respectively. *Z_A_* and *Z_B_* are indicator variables. *Z* is a dummy variable. *Z_A_* = 1 (*Z_B_* = 1) represents that there is carryover effect in Group A (Group B); *Z_A_* = 0 and *Z_B_* = 0 mean no carryover effect in both Group A and Group B. *γ_i_* indicates random effects of the *i*-th subject. *ε_ij_* represents *i*-th subject’s random error for the *j*-th period. The covariance structure for random error of each subject (var(*ε_ij_*)) is equal to Δ. Random subject effect 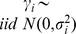. *γ_i_* is independent on *ε_ij_*.

If there is only one subject (*n* = 1), Model 3 reduces to:
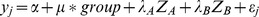
Here, *y_j_* denotes the response value of the *j*-th period, and *ε_j_* represents random error of the *j*-th period.

### Meta-analysis (Model 4)

Each subject of N-of-1 trials is considered as a separate trial (study). A typical method to analyze *n*>1 N-of-1 trials is to use meta-analysis [22]. Meta-analysis combines summary data from each subject to form a weighted average using the method of Der-Simonian and Laird:
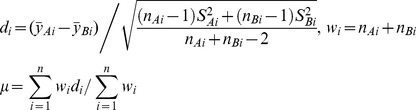
(3)where 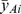 and 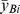 denote the means of Group A and Group B for the *i*-th individual respectively. *S_Ai_* and *S_Bi_* are their corresponding standard deviations respectively. *n_Ai_* and *n_Bi_* are the numbers of *i*-th individual receiving Group A and Group B respectively. *d_i_* represents mean of effect difference for the *i*-th individual, and *w_i_* is its weight. Meta-analysis is not well-defined for N-of-1 trials for *n* = 1 subject.

### Generating Data

The continuous 6-dimensions (for 3-cycles) or 8-dimensions (for 4-cycles) normal distribution data were generated by a multivariate normal random number generator (a SAS Macro) [26], [27] based on mixed effect model. That was, *y_ij_* was generated from multivariate normal distribution according to compound symmetry (CS) structures or first-order autoregressive (AR) structure. Two groups (A or B) of each cycle in each subject were randomly assigned using “Proc Plan” in the SAS 9.2 software. All the subjects in each period were also randomly allocated into Group A or Group B to assure that half of the subjects in each period receive Group A or Group B. The actual response value of each subject was produced according to the allocations. For example, the allocation sequence of the first subject in six periods was BABAAB.

We assumed that carryover effect was caused only by the previous period, and was equal to a certain percentage of the previous treatment effect. We set carryover effect with two levels, no carryover effect (0%) and the presence of it with 20% of the previous treatment effect. That was, *λ_A_* and *λ_B_* was both set to 0% or 20% of the previous treatment effect. For instance, suppose that the allocation sequence of a subject was ABBABA and no carryover effect in the first period. Carryover effect of Group A was added to effect of Group B in the second period, carryover effect of Group B was then added to effect of Group B in the third period, and so on. Carryover rate was defined as carryover effect divided by treatment effect.

### Parameter Setting

The 3-cycles (six periods) and 4-cycles (eight periods) designs of N-of-1 trials were included in the study. The sample size was set to 1, 3, 5, 10, 20 and 30 respectively. Three variance-covariance matrices were assumed as compound symmetry (CS) structures, and two variance-covariance matrices were assumed as first-order autoregressive (AR) structures. All variances in CS or AR structures were set to 1. The covariance (correlation coefficient) of CS structures were set to 0 (CS1), 0.5 (CS2) and 0.8 (CS3) respectively. The covariances of AR1 structure were set to 0.5, 0.5^2^, 0.5^3^, 0.5^4^ and 0.5^5^, with autoregressive coefficient of 0.5. The covariances of AR2 structure were set to 0.8, 0.8^2^, 0.8^3^, 0.8^4^ and 0.8^5^, with autoregressive coefficient of 0.8. The true effect of Group B was equal to 2, while the true effect of Group A was equal to 2, 2.4, 2.6 and 3 respectively. Carryover rate of Group A and Group B were both set to 0% or 20%. The simulation was repeated 3000 times for each parameter setting.

### Analysis and Model Assessment

Data analyses were conducted by the SAS 9.2 software package. To fit the models, covariance structures of Model 2 and Model 3 were set to the structure which was coincident with that of the data generated. Type I error, power, bias (mean error), mean square error (MSE) and percent error (PE) of effect difference were used to assess the performances of the models. Percent error of ME was absolute of ME divided by the true effect difference. Bias, MSE and PE were calculated as follows:
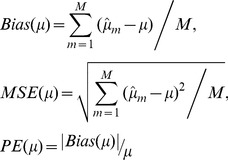
(4)where *μ* represented the true effect difference. 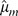
was the estimated effect difference for the *m*-th simulation. *M* was the number of simulation (*M* = 3000).

## Results

### Type I Error

Table 1 presented type I error of the four models for *n* = 1, 3, 5, 10, 20, 30 in 3-cycles N-of-1 trials under five variance-covariance matrices structures with the assumption of what the true effect difference between two groups was 0 (*µ_A_* = *µ_B_* = 2). When *n* = 1, Model 4 was not performed, and the results of Model 1 and Model 2 were the same.

**Table 1:** Type I error of 3-cycles N-of-1 trials (*n* = 1, 3, 5, 10, 20, 30).

Carryover rate	CS1					CS2					CS3					AR1					AR2			
	M1	M2	M3	M4		M1	M2	M3	M4		M1	M2	M3	M4		M1	M2	M3	M4		M1	M2	M3	M4
*n* = 1																								
0%	0.055	0.055	0.050	N/A		0.057	0.057	0.049	N/A		0.059	0.059	0.050	N/A		0.042	0.042	0.027	N/A		0.048	0.048	0.021	N/A
20%	0.056	0.056	0.050	N/A		0.051	0.051	0.049	N/A		0.053	0.053	0.050	N/A		0.036	0.036	0.027	N/A		0.041	0.041	0.021	N/A
*n* = 3																								
0%	0.061	0.004	0.062	0.081		0.059	0.006	0.054	0.086		0.060	0.005	0.054	0.086		0.035	0.006	0.036	0.035		0.041	0.003	0.032	0.021
20%	0.058	0.004	0.062	0.079		0.057	0.004	0.054	0.080		0.053	0.001	0.054	0.068		0.037	0.005	0.036	0.030		0.033	0.001	0.032	0.015
*n* = 5																								
0%	0.042	0.013	0.045	0.069		0.044	0.012	0.043	0.067		0.043	0.012	0.042	0.066		0.025	0.016	0.020	0.020		0.031	0.018	0.016	0.013
20%	0.042	0.012	0.045	0.066		0.044	0.010	0.043	0.062		0.040	0.008	0.042	0.052		0.023	0.014	0.020	0.019		0.023	0.009	0.016	0.015
*n* = 10																								
0%	0.047	0.029	0.061	0.070		0.048	0.029	0.058	0.059		0.047	0.030	0.057	0.057		0.031	0.031	0.029	0.032		0.041	0.032	0.022	0.021
20%	0.046	0.026	0.061	0.064		0.044	0.026	0.058	0.060		0.039	0.021	0.057	0.050		0.029	0.028	0.029	0.031		0.029	0.023	0.022	0.021
*n* = 20																								
0%	0.054	0.038	0.053	0.045		0.052	0.036	0.053	0.042		0.052	0.038	0.054	0.041		0.033	0.033	0.028	0.032		0.038	0.036	0.022	0.018
20%	0.049	0.035	0.053	0.044		0.046	0.032	0.053	0.040		0.038	0.025	0.054	0.036		0.026	0.030	0.028	0.031		0.025	0.027	0.022	0.017
*n* = 30																								
0%	0.053	0.044	0.053	0.036		0.052	0.043	0.049	0.030		0.051	0.044	0.050	0.032		0.029	0.044	0.021	0.032		0.029	0.044	0.015	0.020
20%	0.051	0.042	0.053	0.030		0.046	0.038	0.049	0.027		0.038	0.031	0.050	0.024		0.024	0.040	0.021	0.026		0.018	0.031	0.015	0.018

Note: M1, M2, M3 and M4 denoted paired t-test (Model 1), mixed effects model of difference (Model 2), mixed effects model (Model 3) and meta-analysis (Model 4) respectively. CS1, CS2 and CS3 represented compound symmetry variance-covariance matrices with covariance of 0, 0.5 and 0.8 respectively. AR1, AR2 denoted first-order autoregressive structure with autoregressive coefficient 0.5 and 0.8 respectively. 0% and 20% meant carryover rate. N/A: Meta-analysis was not available for *n* = 1 subject.

Under three CS structures, Model 1 and Model 3 were consistent with each other and yielded type I error near to 5% (the nominal level). Type I error of Model 2 was less than 5%, while that of Model 4 was far from 5% under three CS structures. Type I error of 4 models was less than 5% under two AR structures.

As carryover rate increased from 0% to 20%, type I error of Model 1, Model 2 and Model 4 slightly reduced, but Model 3 was unaffected.

The results of 4-cycles N-of-1 trials performed as well as that of 3-cycles N-of-1 trials (Table S1).

### Power

The true effect differences were set to 0.4, 0.6 and 1.0 respectively. Table 2 showed the power of 3-cycles N-of-1 trials.

**Table 2:** Power of 3-cycles N-of-1 trials (*n* = 1, 3, 5, 10, 20, 30).

Carryover	Effect	CS1				CS2				CS3					AR1				AR2			
rate	difference	M1	M2	M3	M4	M1	M2	M3	M4	M1	M2	M3	M4		M1	M2	M3	M4	M1	M2	M3	M4
*n* = 1																						
0%	0.4	0.070	0.070	0.058	N/A	0.083	0.083	0.064	N/A	0.112	0.112	0.089	N/A		0.062	0.062	0.036	N/A	0.100	0.100	0.042	N/A
	0.6	0.086	0.086	0.068	N/A	0.108	0.108	0.086	N/A	0.168	0.168	0.131	N/A		0.092	0.092	0.052	N/A	0.160	0.160	0.067	N/A
	1.0	0.124	0.124	0.102	N/A	0.177	0.177	0.139	N/A	0.336	0.336	0.264	N/A		0.171	0.171	0.096	N/A	0.322	0.322	0.156	N/A
20%	0.4	0.070	0.070	0.057	N/A	0.069	0.069	0.062	N/A	0.090	0.090	0.084	N/A		0.056	0.056	0.035	N/A	0.080	0.080	0.039	N/A
	0.6	0.071	0.071	0.065	N/A	0.091	0.091	0.080	N/A	0.122	0.122	0.121	N/A		0.072	0.072	0.047	N/A	0.126	0.126	0.061	N/A
	1.0	0.112	0.112	0.095	N/A	0.150	0.150	0.126	N/A	0.254	0.254	0.239	N/A		0.127	0.127	0.086	N/A	0.239	0.239	0.138	N/A
*n* = 3																						
0%	0.4	0.128	0.009	0.099	0.167	0.202	0.011	0.137	0.246	0.387	0.024	0.244	0.437		0.176	0.010	0.088	0.146	0.379	0.017	0.131	0.277
	0.6	0.216	0.014	0.156	0.263	0.355	0.021	0.229	0.409	0.701	0.050	0.460	0.692		0.339	0.018	0.157	0.310	0.696	0.046	0.276	0.576
	1.0	0.216	0.014	0.156	0.505	0.743	0.056	0.504	0.732	0.983	0.189	0.830	0.895		0.754	0.056	0.379	0.690	0.987	0.183	0.630	0.901
20%	0.4	0.111	0.008	0.099	0.140	0.159	0.010	0.137	0.201	0.278	0.012	0.244	0.338		0.131	0.008	0.088	0.121	0.262	0.009	0.131	0.213
	0.6	0.178	0.011	0.156	0.217	0.284	0.013	0.229	0.336	0.541	0.027	0.460	0.573		0.261	0.012	0.157	0.243	0.535	0.023	0.276	0.446
	1.0	0.386	0.019	0.314	0.433	0.625	0.036	0.504	0.630	0.929	0.089	0.830	0.861		0.617	0.040	0.379	0.562	0.933	0.088	0.630	0.818
*n* = 5																						
0%	0.4	0.167	0.022	0.131	0.192	0.292	0.043	0.211	0.309	0.633	0.117	0.443	0.550		0.276	0.047	0.132	0.238	0.641	0.135	0.241	0.502
	0.6	0.327	0.049	0.245	0.337	0.584	0.105	0.408	0.526	0.928	0.281	0.774	0.729		0.589	0.102	0.292	0.504	0.948	0.310	0.544	0.816
	1.0	0.721	0.151	0.550	0.625	0.950	0.317	0.821	0.755	1.000	0.767	0.988	0.834		0.966	0.319	0.692	0.841	1.000	0.750	0.921	0.893
20%	0.4	0.135	0.020	0.131	0.166	0.231	0.031	0.211	0.247	0.470	0.059	0.443	0.439		0.209	0.032	0.132	0.182	0.474	0.075	0.241	0.369
	0.6	0.266	0.036	0.245	0.280	0.461	0.065	0.408	0.442	0.823	0.156	0.774	0.672		0.458	0.074	0.292	0.395	0.840	0.179	0.544	0.705
	1.0	0.612	0.105	0.550	0.554	0.879	0.219	0.821	0.715	0.999	0.537	0.988	0.825		0.904	0.220	0.692	0.781	0.998	0.549	0.921	0.880
*n* = 10																						
0%	0.4	0.323	0.100	0.250	0.254	0.559	0.172	0.410	0.419	0.913	0.407	0.775	0.593		0.556	0.173	0.296	0.460	0.918	0.388	0.526	0.773
	0.6	0.609	0.194	0.474	0.431	0.885	0.367	0.734	0.576	0.999	0.772	0.977	0.661		0.902	0.352	0.595	0.750	1.000	0.740	0.863	0.855
	1.0	0.961	0.494	0.865	0.614	1.000	0.819	0.988	0.668	1.000	0.996	1.000	0.795		1.000	0.795	0.956	0.823	1.000	0.985	0.997	0.832
20%	0.4	0.260	0.080	0.250	0.208	0.443	0.126	0.410	0.342	0.807	0.268	0.775	0.542		0.443	0.125	0.296	0.245	0.820	0.258	0.526	0.675
	0.6	0.497	0.148	0.474	0.375	0.795	0.271	0.734	0.524	0.991	0.582	0.977	0.637		0.812	0.264	0.595	0.667	0.992	0.563	0.863	0.826
	1.0	0.908	0.383	0.865	0.583	0.996	0.678	0.988	0.651	1.000	0.970	1.000	0.761		0.998	0.661	0.956	0.805	1.000	0.955	0.997	0.814
*n* = 20																						
0%	0.4	0.562	0.206	0.434	0.301	0.858	0.370	0.699	0.413	0.999	0.765	0.974	0.460		0.883	0.363	0.542	0.728	0.999	0.749	0.853	0.881
	0.6	0.899	0.410	0.762	0.417	0.997	0.718	0.962	0.454	1.000	0.983	1.000	0.557		0.999	0.709	0.904	0.815	1.000	0.973	0.995	0.825
	1.0	1.000	0.855	0.993	0.474	1.000	0.992	1.000	0.579	1.000	1.000	1.000	0.744		1.000	0.989	1.000	0.781	1.000	0.999	1.000	0.804
20%	0.4	0.462	0.166	0.434	0.269	0.748	0.283	0.699	0.379	0.986	0.588	0.974	0.461		0.776	0.277	0.542	0.646	0.990	0.591	0.853	0.853
	0.6	0.808	0.326	0.762	0.401	0.979	0.576	0.962	0.458	1.000	0.928	1.000	0.514		0.990	0.569	0.904	0.797	1.000	0.918	0.995	0.807
	1.0	0.999	0.749	0.993	0.470	1.000	0.960	1.000	0.535	1.000	1.000	1.000	0.720		1.000	0.960	1.000	0.763	1.000	0.997	1.000	0.789
*n* = 30																						
0%	0.4	0.753	0.303	0.595	0.277	0.965	0.554	0.873	0.324	1.000	0.922	0.999	0.342		0.978	0.879	0.749	0.807	1.000	0.909	0.967	0.863
	0.6	0.981	0.607	0.914	0.326	1.000	0.896	0.997	0.337	1.000	1.000	1.000	0.502		1.000	0.895	0.984	0.787	1.000	0.999	1.000	0.804
	1.0	1.000	0.967	1.000	0.371	1.000	1.000	1.000	0.530	1.000	1.000	1.000	0.724		1.000	1.000	1.000	0.772	1.000	0.999	1.000	0.792
20%	0.4	0.645	0.244	0.595	0.239	0.910	0.441	0.873	0.323	1.000	0.814	0.999	0.347		0.927	0.440	0.749	0.754	1.000	0.803	0.967	0.824
	0.6	0.940	0.499	0.914	0.330	1.000	0.793	0.997	0.339	1.000	0.992	1.000	0.428		1.000	0.793	0.984	0.770	1.000	0.985	1.000	0.758
	1.0	1.000	0.920	1.000	0.355	1.000	0.997	1.000	0.460	1.000	1.000	1.000	0.684		1.000	0.997	1.000	0.722	1.000	0.999	1.000	0.760

* M1: Model 1; M2: Model 2; M3: Model 3; M4: Model 4. N/A: Meta-analysis was not available for *n* = 1 subject.

When *n* = 1, except for Model 4 (unavailable), three models yielded very low power less than 0.34. The power of Model 1 and Model 2 were the same. In practice, one individual design of N-of-1 trials should not be considered unless the effect size is sufficiently large.

The power of all models was increasing with sample size. When *n* >1, Model 1 yielded the highest power, followed by Model 3 at any setting. The power of Model 4 were greater than that of Model 2 when *n* ≤5. The power of Model 2 were greater than that of Model 4 when *n* ≥10.

Under most situations, CS3 structure yielded higher power than other four structures.

As carryover rate increased from 0% to 20%, the power of Model 3 was unaffected by carryover rate, and the power of Model 1, Model 2 and Model 4 had a slight decline.

4-cycles N-of-1 trials (Table S2) yielded higher power than 3-cycles N-of-1 trials.

### Bias and MSE

Table 3 and Table 4 displayed bias and MSE of 3-cycles N-of-1 trials respectively.

**Table 3:** Bias (PE, %) of 3-cycles N-of-1 trials (*n* = 1, 3, 5, 10, 20, 30).

Carryover	Effect	CS1				CS2				CS3				AR1				AR2			
rate	difference	M1	M2	M3	M4	M1	M2	M3	M4	M1	M2	M3	M4	M1	M2	M3	M4	M1	M2	M3	M4
*n* = 1																					
0%	0.0	−0.003	−0.003	−0.002	N/A	−0.002	−0.002	−0.001	N/A	−0.002	−0.002	0.000	N/A	−0.002	−0.002	0.002	N/A	−0.001	−0.001	0.003	N/A
	0.4	−0.004 (0.9)	−0.004 (0.9)	−0.002 (0.4)	N/A	−0.002 (0.6)	−0.002 (0.6)	−0.001 (0.3)	N/A	−0.002 (0.4)	−0.002 (0.4)	0.000 (0.1)	N/A	−0.002 (0.6)	−0.002 (0.6)	0.002 (0.6)	N/A	−0.001 (0.2)	−0.001 (0.2)	0.003 (0.8)	N/A
	0.6	−0.003 (0.6)	−0.003 (0.6)	−0.002 (0.3)	N/A	−0.002 (0.4)	−0.002 (0.4)	−0.001 (0.1)	N/A	−0.002 (0.3)	−0.002 (0.3)	0.000 (0.1)	N/A	−0.002 (0.4)	−0.002 (0.4)	0.002 (0.4)	N/A	−0.001 (0.2)	−0.001 (0.2)	0.003 (0.5)	N/A
	1.0	−0.003 (0.3)	−0.003 (0.3)	−0.002 (0.2)	N/A	−0.002 (0.2)	−0.002 (0.2)	−0.001 (0.1)	N/A	−0.002 (0.2)	−0.002 (0.2)	0.000 (0.0)	N/A	−0.002 (0.2)	−0.002 (0.2)	0.002 (0.2)	N/A	−0.001 (0.1)	−0.001 (0.1)	0.003 (0.3)	N/A
20%	0.0	−0.003	−0.003	−0.002	N/A	−0.002	−0.002	−0.001	N/A	−0.001	−0.001	0.000	N/A	−0.001	−0.001	0.002	N/A	0.000	0.000	0.003	N/A
	0.4	−0.042 (10.4)	−0.042 (10.4)	−0.022 (5.4)	N/A	−0.041 (10.3)	−0.041 (10.3)	−0.021 (5.2)	N/A	−0.040 (10.1)	−0.040 (10.1)	−0.020 (5.1)	N/A	−0.041 (10.2)	−0.041 (10.2)	−0.017 (4.2)	N/A	−0.040 (10.0)	−0.040 (10.0)	−0.017 (4.1)	N/A
	0.6	−0.062 (10.4)	−0.062 (10.4)	−0.031 (5.2)	N/A	−0.061 (10.2)	−0.061 (10.2)	−0.030 (5.1)	N/A	−0.060 (10.0)	−0.060 (10.0)	−0.030 (5.0)	N/A	−0.061 (10.2)	−0.061 (10.2)	−0.027 (4.6)	N/A	−0.060 (9.9)	−0.060 (9.9)	−0.026 (4.4)	N/A
	1.0	−0.102 (10.2)	−0.102 (10.2)	−0.051 (5.1)	N/A	−0.101 (10.1)	−0.101 (10.1)	−0.050 (5.0)	N/A	−0.100 (10.0)	−0.100 (10.0)	−0.050 (5.0)	N/A	−0.101 (10.1)	−0.101 (10.1)	−0.047 (4.7)	N/A	−0.099 (9.9)	−0.099 (9.9)	−0.046 (4.6)	N/A
*n* = 3																					
0%	0.0	−0.001	−0.073	−0.018	0.005	0.000	−0.002	−0.013	0.005	0.000	−0.001	−0.008	0.006	−0.001	−0.002	−0.012	0.004	−0.001	−0.003	−0.007	−0.002
	0.4	−0.001 (0.2)	−0.003 (0.7)	−0.018 (4.5)	0.108 (27.0)	0.000 (0.1)	−0.002 (0.4)	−0.013 (3.3)	0.317 (79.3)	0.000 (0.1)	−0.001 (0.3)	−0.008 (1.9)	0.733 (183.3)	−0.001 (0.2)	−0.002 (0.5)	−0.012 (3.1)	0.176 (44.1)	−0.001 (0.2)	−0.003 (0.6)	−0.007 (1.8)	0.410 (102.4)
	0.6	−0.001 (0.1)	−0.003 (0.5)	−0.018 (3.0)	0.159 (26.5)	0.000 (0.1)	−0.002 (0.3)	−0.013 (2.2)	0.473 (78.8)	0.000 (0.0)	−0.001 (0.2)	−0.008 (1.3)	1.097 (182.8)	−0.001 (0.1)	−0.002 (0.3)	−0.012 (2.0)	0.263 (43.8)	−0.001 (0.2)	−0.003 (0.4)	−0.007 (1.2)	0.616 (102.6)
	1.0	−0.001 (0.1)	−0.003 (0.3)	−0.018 (1.8)	0.262 (26.2)	0.000 (0.0)	−0.002 (0.2)	−0.013 (1.3)	0.785 (78.5)	0.000 (0.0)	−0.001 (0.1)	−0.008 (0.8)	1.824 (182.4)	−0.001 (0.1)	−0.002 (0.2)	−0.012 (1.2)	0.436 (43.6)	−0.001 (0.1)	−0.003 (0.3)	−0.007 (0.7)	1.028 (102.8)
20%	0.0	−0.001	−0.003	−0.018	0.007	−0.001	−0.002	−0.013	0.006	0.000	−0.001	−0.008	0.003	−0.001	−0.002	−0.012	0.014	−0.001	−0.003	−0.007	0.024
	0.4	−0.042 (10.4)	−0.043 (10.8)	−0.018 (4.4)	0.049 (12.2)	−0.041(10.2)	−0.042 (10.5)	−0.013 (3.3)	0.219 (54.8)	−0.041 (10.2)	−0.041 (10.3)	−0.008 (1.9)	0.528 (131.9)	−0.041 (10.3)	−0.042 (10.5)	−0.012 (3.1)	0.116 (29.1)	−0.041 (10.4)	−0.043 (10.7)	−0.007 (1.8)	0.323 (80.8)
	0.6	−0.062 (10.3)	−0.063 (10.5)	−0.018 (3.0)	0.068 (11.3)	−0.061(10.2)	−0.062 (10.4)	−0.013 (2.2)	0.321 (53.5)	−0.061 (10.2)	−0.061 (10.2)	−0.008 (1.3)	0.774 (129.1)	−0.061 (10.2)	−0.062 (10.4)	−0.012 (2.0)	0.166 (27.6)	−0.062 (10.3)	−0.063 (10.5)	−0.007 (1.2)	0.466 (77.6)
	1.0	−0.102 (10.2)	−0.103 (10.3)	−0.018 (1.8)	0.102 (10.2)	−0.102(10.2)	−0.102 (10.2)	−0.013 (1.3)	0.513 (51.3)	−0.101 (10.1)	−0.102 (10.2)	−0.008 (0.8)	1.231 (123.1)	−0.102 (10.2)	−0.103 (10.3)	−0.012 (1.2)	0.268 (26.8)	−0.102 (10.2)	−0.103 (10.3)	−0.007 (0.7)	0.732 (73.2)
*n* = 5																					
0%	0.0	−0.003	−0.012	−0.001	−0.006	−0.003	−0.009	0.001	−0.005	−0.002	−0.006	0.000	−0.004	−0.003	−0.010	−0.001	−0.007	0.000	−0.005	0.000	−0.002
	0.4	−0.003 (0.9)	−0.012 (3.0)	−0.001 (0.4)	0.100 (24.9)	−0.003 (0.6)	−0.009 (2.3)	0.001 (0.2)	0.307 (76.8)	−0.002 (0.4)	−0.006 (1.5)	0.000 (0.1)	0.722 (180.5)	−0.003 (0.7)	−0.010 (2.4)	−0.001 (0.3)	0.170 (42.4)	0.000 (0.1)	−0.005 (1.2)	0.000 (0.0)	0.412 (103.0)
	0.6	−0.003 (0.6)	−0.012 (2.0)	−0.001 (0.2)	0.152 (25.4)	−0.003 (0.4)	−0.009 (1.5)	0.001 (0.1)	0.463 (77.1)	−0.002 (0.3)	−0.006 (1.0)	0.000 (0.1)	1.085 (180.8)	−0.003 (0.5)	−0.010 (1.6)	−0.001 (0.2)	0.258 (43.0)	0.000 (0.1)	−0.005 (0.8)	0.000 (0.0)	0.619 (103.2)
	1.0	−0.003 (0.3)	−0.012 (1.2)	−0.001 (0.1)	0.258 (25.8)	−0.003 (0.3)	−0.009 (0.9)	0.001 (0.1)	0.775 (77.5)	−0.002 (0.2)	−0.006 (0.6)	0.000 (0.0)	1.811 (181.1)	−0.003 (0.3)	−0.010 (1.0)	−0.001 (0.1)	0.435 (43.5)	0.000 (0.0)	−0.005 (0.5)	0.000 (0.0)	1.033 (103.3)
20%	0.0	−0.003	−0.012	−0.001	−0.005	−0.002	−0.009	0.001	0.000	−0.001	−0.006	0.000	0.001	−0.002	−0.010	−0.001	0.005	0.000	−0.005	0.000	0.019
	0.4	−0.043 (10.8)	−0.053 (13.2)	−0.001 (0.4)	0.042 (10.4)	−0.042 (10.6)	−0.050 (12.5)	0.001 (0.2)	0.216 (54.0)	−0.042 (10.4)	−0.047 (11.6)	0.000 (0.1)	0.526 (131.5)	−0.043 (10.7)	−0.050 (12.6)	−0.001 (0.3)	0.113 (28.3)	−0.040 (10.1)	−0.047 (11.8)	0.000 (0.0)	0.318 (79.5)
	0.6	−0.064 (10.6)	−0.073 (12.2)	−0.001 (0.2)	0.063 (10.5)	−0.063 (10.5)	−0.070 (11.7)	0.001 (0.1)	0.319 (53.1)	−0.062 (10.3)	−0.067 (11.2)	0.000 (0.1)	0.772 (128.8)	−0.063 (10.5)	−0.071 (11.8)	−0.001 (0.2)	0.164 (27.4)	−0.061 (10.1)	−0.067 (11.2)	0.000 (0.0)	0.461 (76.8)
	1.0	−0.104 (10.4)	−0.114 (11.4)	−0.001 (0.1)	0.100 (10.0)	−0.103 (10.3)	−0.111 (11.1)	0.001 (0.1)	0.511 (51.1)	−0.102 (10.2)	−0.108 (10.8)	0.000 (0.0)	1.223 (122.3)	−0.103 (10.3)	−0.112 (11.2)	−0.001 (0.1)	0.258 (25.8)	−0.101 (10.1)	−0.107 (10.7)	0.000 (0.0)	0.725 (72.5)
*n* = 10																					
0%	0.0	−0.002	0.000	0.002	−0.003	−0.001	0.000	0.001	−0.002	−0.001	0.000	0.001	−0.002	−0.001	−0.002	0.004	−0.002	−0.001	−0.003	0.002	−0.001
	0.4	−0.002 (0.5)	0.000 (0.1)	0.002 (0.6)	0.103 (25.7)	−0.001 (0.3)	0.000 (0.0)	0.001 (0.3)	0.313 (78.1)	−0.001 (0.2)	0.000 (0.0)	0.001 (0.1)	0.728 (182.1)	−0.001 (0.3)	−0.002 (0.6)	0.004 (1.1)	0.175 (43.6)	−0.001 (0.3)	−0.003 (0.8)	0.002 (0.4)	0.418 (104.5)
	0.6	−0.002 (0.3)	0.000 (0.0)	0.002 (0.4)	0.156 (26.0)	−0.001 (0.2)	0.000 (0.0)	0.001 (0.2)	0.470 (78.3)	−0.001 (0.1)	0.000 (0.0)	0.001 (0.1)	1.093 (182.2)	−0.001 (0.2)	−0.002 (0.4)	0.004 (0.7)	0.263 (43.8)	−0.001 (0.2)	−0.003 (0.5)	0.002 (0.3)	0.627 (104.6)
	1.0	−0.002 (0.2)	0.000 (0.0)	0.002 (0.2)	0.262 (26.2)	−0.001 (0.1)	0.000 (0.0)	0.001 (0.1)	0.785 (78.5)	−0.001 (0.1)	0.000 (0.0)	0.001 (0.1)	1.823 (182.3)	−0.001 (0.1)	−0.002 (0.2)	0.004 (0.4)	0.439 (43.9)	−0.001 (0.1)	−0.003 (0.3)	0.002 (0.2)	1.046 (104.6)
20%	0.0	−0.002	0.000	0.002	−0.001	−0.001	0.000	0.001	−0.001	−0.001	0.000	0.001	0.000	−0.001	0.000	0.004	0.010	−0.001	0.002	0.002	0.026
	0.4	−0.043 (10.9)	−0.042 (10.6)	0.002 (0.6)	0.044 (11.0)	−0.043 (10.7)	−0.042 (10.6)	0.001 (0.3)	0.218 (54.5)	−0.042 (10.6)	−0.042 (10.6)	0.001 (0.1)	0.527 (131.8)	−0.043 (10.7)	−0.042 (10.5)	0.004 (1.1)	0.102 (25.4)	−0.043 (10.7)	−0.041 (10.1)	0.002 (0.4)	0.323 (80.6)
	0.6	−0.064 (10.7)	−0.064 (10.6)	0.002 (0.4)	0.065 (10.8)	−0.064 (10.6)	−0.064 (10.6)	0.001 (0.2)	0.323 (53.8)	−0.063 (10.5)	−0.064 (10.6)	0.001 (0.1)	0.776 (129.4)	−0.064 (10.6)	−0.063 (10.5)	0.004 (0.7)	0.166 (27.7)	−0.063 (10.6)	−0.062 (10.3)	0.002 (0.3)	0.464 (77.3)
	1.0	−0.106 (10.6)	−0.106 (10.6)	0.002 (0.2)	0.104 (10.4)	−0.105 (10.5)	−0.106 (10.6)	0.001 (0.1)	0.522 (52.2)	−0.105 (10.5)	−0.106 (10.6)	0.001 (0.1)	1.236 (123.6)	−0.105 (10.5)	−0.104 (10.4)	0.004 (0.4)	0.260 (26.0)	−0.105 (10.5)	−0.104 (10.4)	0.002 (0.2)	0.729 (72.9)
*n* = 20																					
0%	0.0	−0.003	−0.006	−0.002	−0.002	−0.002	−0.004	−0.002	−0.003	−0.001	−0.003	−0.001	−0.003	−0.002	−0.004	−0.001	−0.002	−0.002	−0.003	−0.001	−0.004
	0.4	−0.003 (0.7)	−0.006 (1.6)	−0.002 (0.5)	0.099 (24.8)	−0.002 (0.5)	−0.004 (1.1)	−0.002 (0.5)	0.307 (76.7)	−0.001 (0.3)	−0.003 (0.7)	−0.001 (0.3)	0.718 (179.6)	−0.002 (0.5)	−0.004 (1.0)	−0.001 (0.3)	0.171 (42.7)	−0.002 (0.4)	−0.003 (0.8)	−0.001 (0.4)	0.411 (102.8)
	0.6	−0.003 (0.5)	−0.006 (1.1)	−0.002 (0.4)	0.150 (25.0)	−0.002 (0.3)	−0.004 (0.7)	−0.002 (0.3)	0.461 (76.9)	−0.001 (0.2)	−0.003 (0.4)	−0.001 (0.2)	1.079 (179.8)	−0.002 (0.3)	−0.004 (0.6)	−0.001 (0.2)	0.257 (42.8)	−0.002 (0.3)	−0.003 (0.5)	−0.001 (0.2)	0.619 (103.1)
	1.0	−0.003 (0.3)	−0.006 (0.6)	−0.002 (0.2)	0.252 (25.2)	−0.002 (0.2)	−0.004 (0.4)	−0.002 (0.2)	0.771 (77.1)	−0.001 (0.1)	−0.003 (0.3)	−0.001 (0.1)	1.800 (180.0)	−0.002 (0.2)	−0.004 (0.4)	−0.001 (0.1)	0.430 (43.0)	−0.002 (0.2)	−0.003 (0.3)	−0.001 (0.1)	1.033 (103.3)
20%	0.0	-0.003	−0.006	−0.002	−0.004	−0.002	−0.004	−0.002	−0.004	−0.001	−0.003	−0.001	−0.003	−0.002	−0.004	−0.001	0.009	−0.002	−0.002	−0.001	0.020
	0.4	−0.044 (11.0)	−0.048 (12.1)	−0.002 (0.5)	0.038 (9.5)	−0.043 (10.7)	−0.046 (11.5)	−0.002 (0.5)	0.211 (52.8)	−0.042 (10.6)	−0.044 (11.1)	−0.001 (0.3)	0.523 (130.8)	−0.043 (10.7)	−0.045 (11.2)	−0.001 (0.3)	0.111 (27.7)	−0.043 (10.7)	−0.044 (11.0)	−0.001 (0.4)	0.316 (78.9)
	0.6	−0.065 (10.8)	−0.069 (11.5)	−0.002 (0.4)	0.058 (9.6)	−0.064 (10.6)	−0.067 (11.2)	−0.002 (0.3)	0.314 (52.4)	−0.063 (10.5)	−0.065 (10.9)	−0.001 (0.2)	0.771 (128.5)	−0.064 (10.6)	−0.066 (11.0)	−0.001 (0.2)	0.159 (26.5)	−0.063 (10.5)	−0.065 (10.8)	−0.001 (0.2)	0.457 (76.1)
	1.0	−0.106 (10.6)	−0.111 (11.1)	−0.002 (0.2)	0.092 (9.2)	−0.105 (10.5)	−0.109 (10.9)	−0.002 (0.2)	0.511 (51.1)	−0.104 (10.4)	−0.107 (10.7)	−0.001 (0.1)	1.226 (122.6)	−0.105 (10.5)	−0.107 (10.7)	−0.001 (0.1)	0.249 (24.9)	−0.104 (10.4)	−0.107 (10.7)	−0.001 (0.1)	0.721 (72.1)
*n* = 30																					
0%	0.0	−0.003	−0.002	−0.005	−0.002	−0.002	−0.001	−0.003	−0.003	−0.001	-0.001	−0.002	−0.003	−0.002	−0.002	−0.003	−0.001	−0.001	−0.001	−0.003	0.001
	0.4	−0.003 (0.8)	−0.002 (0.4)	−0.005 (1.2)	0.100 (24.9)	−0.002 (0.5)	−0.001 (0.3)	−0.003 (0.8)	0.307 (76.8)	−0.001 (0.3)	−0.001 (0.2)	−0.002 (0.5)	0.719 (179.8)	−0.002 (0.5)	0.005 (1.3)	−0.003 (0.8)	0.170 (42.6)	−0.001 (0.3)	−0.001 (0.3)	−0.003 (0.6)	0.415 (103.7)
	0.6	−0.003 (0.5)	−0.002 (0.3)	−0.005 (0.8)	0.151 (25.1)	−0.002 (0.3)	−0.001 (0.2)	−0.003 (0.5)	0.462 (77.0)	−0.001 (0.2)	−0.001 (0.1)	−0.002 (0.3)	1.081 (180.1)	−0.002 (0.4)	−0.002 (0.3)	−0.003 (0.5)	0.256 (42.7)	−0.001 (0.2)	−0.001 (0.2)	−0.003 (0.4)	0.622 (103.7)
	1.0	−0.003 (0.3)	−0.002 (0.2)	−0.005 (0.5)	0.252 (25.2)	−0.002 (0.2)	−0.001 (0.1)	−0.003 (0.3)	0.772 (77.2)	−0.001 (0.1)	−0.001 (0.1)	−0.002 (0.2)	1.803 (180.3)	−0.002 (0.2)	−0.002 (0.2)	−0.003 (0.3)	0.427 (42.7)	−0.001 (0.1)	−0.001 (0.1)	−0.003 (0.3)	1.036 (103.6)
20%	0.0	−0.003	−0.002	−0.005	−0.003	−0.002	−0.001	−0.003	−0.004	−0.001	−0.001	−0.002	−0.003	−0.002	−0.002	−0.003	0.009	−0.001	0.000	−0.003	0.023
	0.4	−0.044 (11.0)	−0.043 (10.8)	−0.005 (1.2)	0.041 (10.2)	−0.043 (10.7)	−0.043 (10.7)	−0.003 (0.8)	0.212 (53.0)	−0.042 (10.5)	−0.042 (10.6)	−0.002 (0.5)	0.524 (130.9)	−0.043 (10.7)	−0.043 (10.8)	−0.003 (0.8)	0.112 (28.0)	−0.042 (10.5)	−0.042 (10.4)	−0.003 (0.6)	0.322 (80.5)
	0.6	−0.064 (10.7)	−0.064 (10.6)	−0.005 (0.8)	0.061 (10.2)	−0.063 (10.5)	−0.063 (10.6)	−0.003 (0.5)	0.316 (52.7)	−0.062 (10.4)	−0.063 (10.5)	−0.002 (0.3)	0.772 (128.7)	−0.063 (10.5)	−0.064 (10.6)	−0.003 (0.5)	0.161 (26.9)	−0.062 (10.4)	−0.062 (10.4)	−0.003 (0.4)	0.464 (77.4)
	1.0	−0.105 (10.5)	−0.105 (10.5)	−0.005 (0.5)	0.098 (9.8)	−0.104 (10.4)	−0.105 (10.5)	−0.003 (0.3)	0.514 (51.4)	−0.103 (10.3)	−0.104 (10.4)	−0.002 (0.2)	1.231 (123.1)	−0.104 (10.4)	−0.105 (10.5)	−0.003 (0.3)	0.253 (25.3)	−0.103 (10.3)	−0.104 (10.4)	−0.003 (0.3)	0.731 (73.1)

M1: Model 1; M2: Model 2; M3: Model 3; M4: Model 4. N/A: Meta-analysis was not available for *n* = 1 subject.

**Table 4:** MSE of 3-cycles N-of-1 trials (*n* = 1, 3, 5, 10, 20, 30).

Carryover	Effect	**CS1**				**CS2**				**CS3**				**AR1**				**AR2**			
rate	**difference**	**M1**	**M2**	**M3**	**M4**	**M1**	**M2**	**M3**	**M4**	**M1**	**M2**	**M3**	**M4**	**M1**	**M2**	**M3**	**M4**	**M1**	**M2**	**M3**	**M4**
***n*** ** = 1**																					
0%	0.0	0.674	0.674	1.191	N/A	0.337	0.337	0.600	N/A	0.135	0.135	0.240	N/A	0.275	0.275	0.623	N/A	0.116	0.116	0.299	N/A
	0.4	0.674	0.674	1.191	N/A	0.337	0.337	0.601	N/A	0.135	0.135	0.240	N/A	0.275	0.275	0.623	N/A	0.116	0.116	0.299	N/A
	0.6	0.674	0.674	1.191	N/A	0.337	0.337	0.600	N/A	0.135	0.135	0.240	N/A	0.275	0.275	0.623	N/A	0.116	0.116	0.299	N/A
	1.0	0.674	0.674	1.191	N/A	0.337	0.337	0.600	N/A	0.135	0.135	0.240	N/A	0.275	0.275	0.623	N/A	0.116	0.116	0.299	N/A
20%	0.0	0.688	0.688	1.191	N/A	0.352	0.352	0.600	N/A	0.151	0.151	0.240	N/A	0.290	0.290	0.623	N/A	0.133	0.133	0.299	N/A
	0.4	0.697	0.697	1.193	N/A	0.358	0.358	0.602	N/A	0.157	0.157	0.242	N/A	0.296	0.296	0.623	N/A	0.138	0.138	0.302	N/A
	0.6	0.699	0.699	1.195	N/A	0.363	0.363	0.604	N/A	0.162	0.162	0.244	N/A	0.301	0.301	0.628	N/A	0.143	0.143	0.304	N/A
	1.0	0.712	0.712	1.202	N/A	0.375	0.375	0.611	N/A	0.174	0.174	0.251	N/A	0.313	0.313	0.635	N/A	0.155	0.155	0.311	N/A
*n* = 3																					
0%	0.0	0.239	0.434	0.461	0.479	0.121	0.330	0.237	1.064	0.049	0.132	0.096	0.501	0.094	0.322	0.287	0.241	0.039	0.120	0.148	0.192
	0.4	0.239	0.660	0.461	0.527	0.121	0.330	0.237	0.668	0.049	0.132	0.096	1.232	0.094	0.322	0.287	0.317	0.039	0.120	0.148	0.421
	0.6	0.239	0.660	0.461	0.576	0.121	0.330	0.237	0.868	0.049	0.132	0.096	2.097	0.094	0.322	0.287	0.402	0.039	0.120	0.148	0.713
	1.0	0.239	0.660	0.461	0.728	0.121	0.330	0.237	1.491	0.049	0.132	0.096	4.827	0.094	0.322	0.287	0.665	0.039	0.120	0.148	1.655
20%	0.0	0.239	0.660	0.461	0.464	0.121	0.330	0.237	0.472	0.050	0.132	0.096	0.458	0.095	0.322	0.287	0.246	0.041	0.120	0.148	0.184
	0.4	0.241	0.664	0.461	0.493	0.124	0.334	0.237	0.548	0.052	0.135	0.096	0.786	0.097	0.325	0.287	0.296	0.043	0.123	0.148	0.349
	0.6	0.244	0.668	0.461	0.522	0.126	0.337	0.237	0.635	0.054	0.138	0.096	1.180	0.100	0.328	0.287	0.359	0.045	0.127	0.148	0.529
	1.0	0.251	0.679	0.461	0.624	0.134	0.347	0.237	0.903	0.062	0.148	0.096	2.336	0.107	0.339	0.287	1.307	0.053	0.137	0.148	1.045
*n* = 5																					
0%	0.0	0.124	0.358	0.202	0.259	0.063	0.176	0.105	0.255	0.025	0.070	0.042	0.260	0.049	0.174	0.119	0.129	0.021	0.078	0.058	0.093
	0.4	0.124	0.358	0.202	0.285	0.063	0.176	0.105	0.373	0.025	0.070	0.042	0.843	0.049	0.174	0.119	0.172	0.021	0.078	0.058	0.304
	0.6	0.124	0.358	0.202	0.318	0.063	0.176	0.105	0.526	0.025	0.070	0.042	1.585	0.049	0.174	0.119	0.232	0.021	0.078	0.058	0.570
	1.0	0.124	0.358	0.202	0.421	0.063	0.176	0.105	1.020	0.025	0.070	0.042	3.970	0.049	0.174	0.119	0.427	0.021	0.078	0.058	1.422
20%	0.0	0.126	0.358	0.202	0.247	0.064	0.176	0.105	0.247	0.026	0.070	0.042	0.240	0.050	0.174	0.119	0.133	0.022	0.078	0.058	0.095
	0.4	0.128	0.360	0.202	0.261	0.066	0.178	0.105	0.313	0.028	0.073	0.042	0.562	0.052	0.176	0.119	0.163	0.024	0.081	0.058	0.240
	0.6	0.130	0.363	0.202	0.278	0.069	0.181	0.105	0.389	0.031	0.075	0.042	0.941	0.055	0.179	0.119	0.198	0.026	0.084	0.058	0.399
	1.0	0.138	0.372	0.202	0.325	0.076	0.190	0.105	0.615	0.038	0.084	0.042	1.956	0.062	0.188	0.119	0.299	0.033	0.093	0.058	0.827
*n* = 10																					
0%	0.0	0.069	0.199	0.109	0.136	0.034	0.099	0.054	0.132	0.014	0.040	0.022	0.132	0.028	0.099	0.061	0.069	0.012	0.041	0.030	0.056
	0.4	0.069	0.199	0.109	0.154	0.034	0.099	0.054	0.245	0.014	0.040	0.022	0.700	0.028	0.099	0.061	0.111	0.012	0.041	0.030	0.253
	0.6	0.069	0.199	0.109	0.177	0.034	0.099	0.054	0.386	0.014	0.040	0.022	1.410	0.028	0.099	0.061	0.162	0.012	0.041	0.030	0.499
	1.0	0.069	0.199	0.109	0.251	0.034	0.099	0.054	0.839	0.014	0.040	0.022	3.685	0.028	0.099	0.061	0.328	0.012	0.041	0.030	1.288
20%	0.0	0.069	0.199	0.109	0.134	0.034	0.099	0.054	0.128	0.014	0.040	0.022	0.117	0.028	0.100	0.061	0.071	0.012	0.041	0.030	0.059
	0.4	0.071	0.200	0.109	0.142	0.036	0.101	0.054	0.186	0.015	0.042	0.022	0.415	0.030	0.102	0.061	0.059	0.014	0.043	0.030	0.179
	0.6	0.073	0.203	0.109	0.151	0.038	0.104	0.054	0.256	0.018	0.044	0.022	0.765	0.032	0.104	0.061	0.118	0.017	0.045	0.030	0.309
	1.0	0.081	0.211	0.109	0.182	0.045	0.111	0.054	0.465	0.025	0.052	0.022	1.762	0.040	0.111	0.061	0.197	0.024	0.053	0.030	0.681
*n* = 20																					
0%	0.0	0.033	0.099	0.050	0.065	0.016	0.049	0.026	0.066	0.007	0.020	0.010	0.066	0.013	0.047	0.028	0.034	0.006	0.020	0.014	0.026
	0.4	0.033	0.099	0.050	0.079	0.016	0.049	0.026	0.167	0.007	0.020	0.010	0.599	0.013	0.047	0.028	0.072	0.006	0.020	0.014	0.205
	0.6	0.033	0.099	0.050	0.096	0.016	0.049	0.026	0.295	0.007	0.020	0.010	1.270	0.013	0.047	0.028	0.118	0.006	0.020	0.014	0.431
	1.0	0.033	0.099	0.050	0.152	0.016	0.049	0.026	0.705	0.007	0.020	0.010	3.417	0.013	0.047	0.028	0.265	0.006	0.020	0.014	1.159
20%	0.0	0.033	0.099	0.050	0.065	0.016	0.049	0.026	0.064	0.007	0.020	0.010	0.062	0.013	0.047	0.028	0.034	0.006	0.020	0.014	0.026
	0.4	0.035	0.101	0.050	0.067	0.018	0.051	0.026	0.112	0.008	0.022	0.010	0.347	0.015	0.050	0.028	0.050	0.007	0.022	0.014	0.134
	0.6	0.037	0.104	0.050	0.073	0.021	0.054	0.026	0.173	0.011	0.024	0.010	0.681	0.017	0.052	0.028	0.068	0.010	0.024	0.014	0.252
	1.0	0.044	0.112	0.050	0.088	0.028	0.062	0.026	0.356	0.018	0.032	0.010	1.621	0.024	0.060	0.028	0.118	0.017	0.032	0.014	0.594
*n* = 30																					
0%	0.0	0.022	0.065	0.032	0.043	0.011	0.032	0.016	0.043	0.004	0.013	0.007	0.043	0.009	0.033	0.018	0.022	0.004	0.013	0.009	0.018
	0.4	0.022	0.065	0.032	0.055	0.011	0.032	0.016	0.141	0.004	0.013	0.007	0.571	0.009	0.009	0.018	0.054	0.004	0.013	0.009	0.197
	0.6	0.022	0.065	0.032	0.071	0.011	0.032	0.016	0.266	0.004	0.013	0.007	1.235	0.009	0.033	0.018	0.095	0.004	0.013	0.009	0.421
	1.0	0.022	0.065	0.032	0.120	0.011	0.032	0.016	0.666	0.004	0.013	0.007	3.362	0.009	0.033	0.018	0.224	0.004	0.013	0.009	1.138
20%	0.0	0.022	0.065	0.032	0.043	0.011	0.032	0.016	0.042	0.004	0.013	0.007	0.040	0.009	0.033	0.018	0.022	0.004	0.013	0.009	0.018
	0.4	0.024	0.067	0.032	0.047	0.013	0.034	0.016	0.090	0.006	0.015	0.007	0.320	0.011	0.035	0.018	0.037	0.006	0.015	0.009	0.128
	0.6	0.026	0.069	0.032	0.051	0.015	0.037	0.016	0.149	0.008	0.017	0.007	0.652	0.013	0.037	0.018	0.054	0.008	0.017	0.009	0.247
	1.0	0.033	0.076	0.032	0.064	0.022	0.044	0.016	0.325	0.015	0.024	0.007	1.597	0.020	0.044	0.018	0.102	0.014	0.024	0.009	0.587

M1: Model 1; M2: Model 2; M3: Model 3; M4: Model 4. N/A: Meta-analysis was not available for *n* = 1 subject.

In the absence of carryover effect, bias of Model 1, Model 2 and Model 3 was minuscule (near 0), while bias of Model 4 was larger.

Carryover rate had an impact on bias of Model 1, Model 2 and Model 4 except Model 3. In the presence of 20% carryover effect, bias of Model 3 was the smallest, followed by Model 1, Model 2 and Model 4. PE of Model 3 was equal to 0. PE of Model 1, Model 2 was equal to 10%, half of carryover rate. PE of Model 4 was the biggest.

MSE of Model 1 was the smallest, followed by Model 1, Model 2 and Model 4, whether there was carryover effect or not. Carryover rate had an impact on MSE of Model 1, Model 2 and Model 4 except Model 3.

Bias of 4-cycles N-of-1 trials was similar with that of 3-cycles N-of-1 trials, with smaller MSE (Table S3 and Table S4).

### Examples

The first example was concerned with Ornithine transcarbamylase deficiency (OTCD) of 3-cycles N-of-1 trials [28]. A 48-year-old patient with OTCD was treated by either L-arginine capsules (test group) or placebo capsules (control group) for weekly periods. The patient and physicians were blinded to treatment. Plasma glutamine as an endpoint was measured about 3 pm on 7^th^ day. The mean differences of plasma glutamine between two groups were estimated as −137.7 (*P* = 0.078, 95% CI: −38.4, 313.8) by Model 1 (or Model 2) and −118.7 (*P* = 0.129, 95% CI: −63.0, 300.3) by Model 3. The results showed that Model 3 yielded larger *P* value and wider confidence interval than Model 1 (or Model 2).

The second example was based on idiopathic chronic fatigue of 3-cycles N-of-1 trials [29]. N-of-1 double-blinded, randomized trials were performed on four physicians who complained of chronic fatigue. Each physician received three pairs of treatments comprising 4 weeks of Spirulina platensis (test group) and 4 weeks of placebo (control group), with 2 weeks washout time. Severity of fatigue was measured on a 10-point scale daily during the second half (weeks 3 and 4) of each period. The outcome was the mean fatigue scores in the same period. The data was re-analyzed using the four models. The effect difference estimators of fatigue between two groups were −0.11 (*P* = 0.485, 95% CI: −0.43, 0.22) by Model 1, −0.15 (*P* = 0.591, 95% CI: −0.92, 0.63) by Model 2, −0.15 (*P* = 0.604, 95% CI: −0.86, 0.57) by Model 3, and −0.15 (95% CI: −2.31, 2.01) by Model 4. 95% CI and *P* value of Model 1 was the smallest. The second smallest 95% CI was Model 3, followed by Model 2 and Model 4. The results were consistent with that of simulation study.

## Discussions

We conducted a simulation study to compare four models of N-of-1 trials. The process of generating data was based on five variance-covariance matrices with parameter setting of cycle number, carryover effect and sample size. The performance of four models were assessed by type I error, power, bias and MSE.

Paired t-test was recommended to use for normally distributed data of N-of-1 trials irrespective with or without carryover effect. Comparing with other three models, paired t-test yielded the highest power almost in all situations, with type I error nearer to the nominal level. Paired t-test had the smallest MSE almost in all situations, and its bias was comparable with mixed effects model and mixed effects model of difference. The real examples from Hackett et al [28] and Baicus et al [29] showed that the results were consistent with the simulation study. In addition; paired t-test was simple to apply, and the result could be explained easily. However, mixed effects model of difference, meta-analysis with the method of Der-Simonian and Laird were not suitable to analyze the data of N-of-1 trials, due to their low power, large bias and MSE.

Though carryover effect may exist in cross-over studies as like N-of-1 trials theoretically, it is not plausible in practice owing to enough washout period [30], [31]. Based on our simulation results, 20% carryover effect has a little influence on the estimation compared with the absence of carryover effect. Therefore, carryover effect is usually ignored in clinical trials.

Mixed effects model was also recommended to use for the data of N-of-1 trials if there was carryover effect. Mixed effects model could deal with carryover effect flexibly, and separated carryover effect from treatment effect in *n*≠1 N-of-1 trials. The results of 20% carryover effect showed that bias in mixed effects model was approximate equal to 0. However, mixed effects model took into account period effect, carryover effect, and random individual effect, resulting in a larger standard deviation for the estimators of effect difference. So mixed effects model yielded bigger MSE. As carryover rate increased from 0% to 20%, the power of mixed effects model was close to (lower than) that of paired t-test. In addition, mixed effects model had a variety of advantages, such as dealing with the missing data, considering numerous variance-covariance structures, and adding new covariables according to the different conditions. For example, some diseases might improve (such as self-healing disease) or deteriorate (such as cancer) over time. We could add a covariate of time into mixed effects model.

As sample size increased, type I error and bias of four models remain unchanged, MSE of four models gradually reduced, and the power of all the models (except meta-analysis) increased. The 3-cycles and 4-cycles N-of-1 trials had the comparable results in type I error and Bias. The 4-cycles N-of-1 trial yielded higher power and smaller MSE than 3-cycles N-of-1 trial because more periods led to the effect such as increasing sample size.

The simulation study essentially assumed that variance of within-subject was equal. We assumed that the variance of within-subject is not equal, for instance, with a sample size of 20, within-subject variance of 10 subjects being 1 and that of the other 10 subjects being 2. The simulation results show that type I error and bias of unequal variance of within-subject are similar to that of equal variance. The power of unequal variance is less than the corresponding power of equal variance. MSE of unequal variance is larger than that of the equal variance due to the bigger variance of raw data. These signify that unequal variance of within-subject could reduce the power but does not affect type I error or bias.

There were several limitations in this study (1) The simulation study considered only the compound symmetry and first-order autoregressive variance-covariance matrices. It did not take into account other variance-covariance matrices. In practice, the correlation between different times should be more complex rather than completely the same or autoregressive. (2) Unbalanced (different cycles) N-of-1 trials which are also applied in practice were not considered in our study. (3) The simulation study did not consider missing data. (4) The simulation assumed that each subject had one measurement (after treatment) per period, thus not accounting for potential information from repeated measurements within each period.

In conclusion, paired t-test was simple and easy to apply, with better statistical performance. It was recommended to use for normally distributed data of N-of-1 trials. Mixed effects model provided an alternative when there was carryover effect.

## Supporting Information

Table S1 **Type I error of 4-cycles N-of-1 trials (** ***n*** **= 1, 3, 5, 10, 20, 30).**
(DOC)

Table S2 **Power of 4-cycles N-of-1 trials (** ***n*** **= 1, 3, 5, 10, 20, 30).**
(DOC)

Table S3 **Bias (PE, %) of 4-cycles N-of-1 trials (** ***n*** **= 1, 3, 5, 10, 20, 30).**
(DOC)

Table S4 **MSE of 4-cycles N-of-1 trials (** ***n*** **= 1, 3, 5, 10, 20, 30).**
(DOC)
