# Supplementary material for: A Comparison of Four Methods for the Analysis of N-of-1 Trials
Source: PLoS One. 2014 Feb 4;9(2):e87752. doi: 10.1371/journal.pone.0087752 (PMC3913644; doi:10.1371/journal.pone.0087752)
Supplement: Table S1 — Type I error of 4-cycles N-of-1 trials ( n = 1, 3, 5, 10, 20, 30). (DOC) [file pone.0087752.s001.doc]

**Table S1. Type I error of 4-cycles N-of-1 trials (*n*=1, 3, 5, 10, 20, 30)**.

| Carryover rate | CS1 | | | |  | CS2 | | | |  | CS3 | | | |  | AR1 | | | |  | AR2 | | | |
| --- | --- | --- | --- | --- | --- | --- | --- | --- | --- | --- | --- | --- | --- | --- | --- | --- | --- | --- | --- | --- | --- | --- | --- | --- |
|  | M1 | M2 | M3 | M4 |  | M1 | M2 | M3 | M4 |  | M1 | M2 | M3 | M4 |  | M1 | M2 | M3 | M4 |  | M1 | M2 | M3 | M4 |
| *n*=1 |  |  |  |  |  |  |  |  |  |  |  |  |  |  |  |  |  |  |  |  |  |  |  |  |
| 0% | 0.056 | 0.056 | 0.049 | N/A |  | 0.057 | 0.057 | 0.049 | N/A |  | 0.057 | 0.057 | 0.051 | N/A |  | 0.031 | 0.031 | 0.024 | N/A |  | 0.042 | 0.042 | 0.018 | N/A |
| 20% | 0.053 | 0.053 | 0.049 | N/A |  | 0.049 | 0.049 | 0.049 | N/A |  | 0.047 | 0.047 | 0.051 | N/A |  | 0.031 | 0.031 | 0.024 | N/A |  | 0.038 | 0.038 | 0.018 | N/A |
| *n*=3 |  |  |  |  |  |  |  |  |  |  |  |  |  |  |  |  |  |  |  |  |  |  |  |  |
| 0% | 0.052 | 0.002 | 0.060 | 0.075 |  | 0.054 | 0.003 | 0.055 | 0.077 |  | 0.054 | 0.002 | 0.055 | 0.076 |  | 0.028 | 0.002 | 0.024 | 0.019 |  | 0.028 | 0.002 | 0.019 | 0.010 |
| 20% | 0.597 | 0.022 | 0.437 | 0.077 |  | 0.048 | 0.002 | 0.055 | 0.072 |  | 0.043 | 0.002 | 0.055 | 0.068 |  | 0.028 | 0.002 | 0.024 | 0.018 |  | 0.023 | 0.001 | 0.019 | 0.007 |
| *n*=5 |  |  |  |  |  |  |  |  |  |  |  |  |  |  |  |  |  |  |  |  |  |  |  |  |
| 0% | 0.046 | 0.016 | 0.049 | 0.061 |  | 0.046 | 0.015 | 0.045 | 0.060 |  | 0.046 | 0.015 | 0.044 | 0.061 |  | 0.020 | 0.014 | 0.020 | 0.011 |  | 0.026 | 0.015 | 0.012 | 0.008 |
| 20% | 0.042 | 0.014 | 0.049 | 0.055 |  | 0.039 | 0.012 | 0.045 | 0.056 |  | 0.035 | 0.009 | 0.044 | 0.052 |  | 0.020 | 0.014 | 0.020 | 0.013 |  | 0.022 | 0.01 | 0.012 | 0.006 |
| *n*=10 |  |  |  |  |  |  |  |  |  |  |  |  |  |  |  |  |  |  |  |  |  |  |  |  |
| 0% | 0.044 | 0.025 | 0.058 | 0.061 |  | 0.045 | 0.026 | 0.055 | 0.059 |  | 0.046 | 0.027 | 0.055 | 0.059 |  | 0.026 | 0.026 | 0.019 | 0.015 |  | 0.030 | 0.026 | 0.008 | 0.007 |
| 20% | 0.042 | 0.024 | 0.058 | 0.056 |  | 0.041 | 0.023 | 0.055 | 0.058 |  | 0.034 | 0.02 | 0.055 | 0.049 |  | 0.021 | 0.024 | 0.019 | 0.013 |  | 0.023 | 0.018 | 0.008 | 0.006 |
| *n*=20 |  |  |  |  |  |  |  |  |  |  |  |  |  |  |  |  |  |  |  |  |  |  |  |  |
| 0% | 0.051 | 0.034 | 0.055 | 0.063 |  | 0.049 | 0.035 | 0.052 | 0.061 |  | 0.050 | 0.036 | 0.052 | 0.060 |  | 0.027 | 0.039 | 0.016 | 0.016 |  | 0.029 | 0.035 | 0.009 | 0.007 |
| 20% | 0.048 | 0.033 | 0.055 | 0.064 |  | 0.045 | 0.031 | 0.052 | 0.058 |  | 0.041 | 0.028 | 0.052 | 0.052 |  | 0.024 | 0.036 | 0.016 | 0.015 |  | 0.020 | 0.027 | 0.009 | 0.006 |
| *n*=30 |  |  |  |  |  |  |  |  |  |  |  |  |  |  |  |  |  |  |  |  |  |  |  |  |
| 0% | 0.054 | 0.047 | 0.057 | 0.049 |  | 0.052 | 0.047 | 0.056 | 0.050 |  | 0.051 | 0.047 | 0.057 | 0.047 |  | 0.028 | 0.052 | 0.021 | 0.023 |  | 0.028 | 0.045 | 0.011 | 0.009 |
| 20% | 0.052 | 0.045 | 0.057 | 0.053 |  | 0.047 | 0.042 | 0.056 | 0.048 |  | 0.043 | 0.039 | 0.057 | 0.047 |  | 0.026 | 0.047 | 0.021 | 0.022 |  | 0.023 | 0.038 | 0.011 | 0.007 |

Note: M1, M2, M3 and M4 denoted paired t-test (Model 1), mixed effects model of difference (Model 2), mixed effects model (Model 3) and meta-analysis (Model 4) respectively. CS1, CS2 and CS3 represented compound symmetry variance-covariance matrices with covariance of 0, 0.5 and 0.8 respectively. AR1, AR2 denoted first-order autoregressive structure with autoregressive coefficient 0.5 and 0.8 respectively. 0% and 20% meant carryover rate. N/A: Meta-analysis was not available for *n*=1 subject.
