# Supplementary material for: A Comparison of Four Methods for the Analysis of N-of-1 Trials
Source: PLoS One. 2014 Feb 4;9(2):e87752. doi: 10.1371/journal.pone.0087752 (PMC3913644; doi:10.1371/journal.pone.0087752)
Supplement: Table S2 — Power of 4-cycles N-of-1 trials ( n = 1, 3, 5, 10, 20, 30). (DOC) [file pone.0087752.s002.doc]

**Table S2. Power of 4-cycles N-of-1 trials (*n***=1, 3, 5, 10, 20, 30).

| Carryover | Effect | CS1 | | | |  | CS2 | | | |  | CS3 | | | |  | AR1 | | | |  | AR2 | | | |
| --- | --- | --- | --- | --- | --- | --- | --- | --- | --- | --- | --- | --- | --- | --- | --- | --- | --- | --- | --- | --- | --- | --- | --- | --- | --- |
| rate | difference | M1 | M2 | M3 | M4 |  | M1 | M2 | M3 | M4 |  | M1 | M2 | M3 | M4 |  | M1 | M2 | M3 | M4 |  | M1 | M2 | M3 | M4 |
| *n*=1 |  |  |  |  |  |  |  |  |  |  |  |  |  |  |  |  |  |  |  |  |  |  |  |  |  |
| 0% | 0.4 | 0.071 | 0.071 | 0.064 | N/A |  | 0.089 | 0.089 | 0.082 | N/A |  | 0.148 | 0.148 | 0.127 | N/A |  | 0.061 | 0.061 | 0.042 | N/A |  | 0.133 | 0.133 | 0.049 | N/A |
|  | 0.6 | 0.095 | 0.095 | 0.081 | N/A |  | 0.137 | 0.137 | 0.118 | N/A |  | 0.267 | 0.267 | 0.212 | N/A |  | 0.098 | 0.098 | 0.062 | N/A |  | 0.247 | 0.247 | 0.096 | N/A |
|  | 1.0 | 0.173 | 0.173 | 0.138 | N/A |  | 0.292 | 0.292 | 0.229 | N/A |  | 0.567 | 0.567 | 0.463 | N/A |  | 0.213 | 0.213 | 0.127 | N/A |  | 0.548 | 0.548 | 0.258 | N/A |
| 20% | 0.4 | 0.068 | 0.068 | 0.064 | N/A |  | 0.081 | 0.081 | 0.079 | N/A |  | 0.122 | 0.122 | 0.121 | N/A |  | 0.061 | 0.061 | 0.042 | N/A |  | 0.103 | 0.103 | 0.047 | N/A |
|  | 0.6 | 0.084 | 0.084 | 0.079 | N/A |  | 0.118 | 0.118 | 0.113 | N/A |  | 0.206 | 0.206 | 0.201 | N/A |  | 0.098 | 0.098 | 0.062 | N/A |  | 0.185 | 0.185 | 0.091 | N/A |
|  | 1.0 | 0.145 | 0.145 | 0.132 | N/A |  | 0.230 | 0.230 | 0.216 | N/A |  | 0.436 | 0.436 | 0.442 | N/A |  | 0.213 | 0.213 | 0.127 | N/A |  | 0.416 | 0.416 | 0.238 | N/A |
| *n*=3 |  |  |  |  |  |  |  |  |  |  |  |  |  |  |  |  |  |  |  |  |  |  |  |  |  |
| 0% | 0.4 | 0.158 | 0.005 | 0.123 | 0.197 |  | 0.252 | 0.008 | 0.171 | 0.289 |  | 0.518 | 0.018 | 0.350 | 0.544 |  | 0.225 | 0.007 | 0.095 | 0.167 |  | 0.504 | 0.016 | 0.144 | 0.302 |
|  | 0.6 | 0.279 | 0.008 | 0.202 | 0.313 |  | 0.477 | 0.017 | 0.326 | 0.506 |  | 0.848 | 0.042 | 0.634 | 0.815 |  | 0.459 | 0.015 | 0.195 | 0.368 |  | 0.863 | 0.037 | 0.341 | 0.667 |
|  | 1.0 | 0.597 | 0.022 | 0.437 | 0.614 |  | 0.882 | 0.049 | 0.678 | 0.842 |  | 0.997 | 0.165 | 0.940 | 0.954 |  | 0.897 | 0.051 | 0.520 | 0.823 |  | 0.999 | 0.162 | 0.744 | 0.945 |
| 20% | 0.4 | 0.597 | 0.022 | 0.437 | 0.165 |  | 0.211 | 0.006 | 0.171 | 0.240 |  | 0.396 | 0.010 | 0.350 | 0.428 |  | 0.176 | 0.005 | 0.095 | 0.132 |  | 0.375 | 0.008 | 0.163 | 0.232 |
|  | 0.6 | 0.234 | 0.008 | 0.202 | 0.260 |  | 0.382 | 0.012 | 0.326 | 0.419 |  | 0.714 | 0.020 | 0.634 | 0.701 |  | 0.366 | 0.001 | 0.195 | 0.297 |  | 0.736 | 0.017 | 0.399 | 0.524 |
|  | 1.0 | 0.501 | 0.016 | 0.437 | 0.538 |  | 0.788 | 0.031 | 0.678 | 0.758 |  | 0.986 | 0.084 | 0.940 | 0.922 |  | 0.806 | 0.030 | 0.519 | 0.697 |  | 0.988 | 0.079 | 0.818 | 0.900 |
| *n*=5 |  |  |  |  |  |  |  |  |  |  |  |  |  |  |  |  |  |  |  |  |  |  |  |  |  |
| 0% | 0.4 | 0.211 | 0.031 | 0.169 | 0.231 |  | 0.394 | 0.051 | 0.289 | 0.396 |  | 0.761 | 0.112 | 0.609 | 0.687 |  | 0.379 | 0.051 | 0.162 | 0.280 |  | 0.778 | 0.116 | 0.280 | 0.550 |
|  | 0.6 | 0.433 | 0.054 | 0.337 | 0.431 |  | 0.723 | 0.101 | 0.563 | 0.657 |  | 0.984 | 0.288 | 0.909 | 0.854 |  | 0.754 | 0.107 | 0.378 | 0.623 |  | 0.988 | 0.283 | 0.644 | 0.895 |
|  | 1.0 | 0.849 | 0.146 | 0.716 | 0.766 |  | 0.990 | 0.324 | 0.931 | 0.866 |  | 1.000 | 0.758 | 0.999 | 0.930 |  | 0.998 | 0.329 | 0.821 | 0.932 |  | 1.000 | 0.751 | 0.972 | 0.945 |
| 20% | 0.4 | 0.169 | 0.024 | 0.169 | 0.199 |  | 0.308 | 0.037 | 0.289 | 0.328 |  | 0.631 | 0.066 | 0.609 | 0.580 |  | 0.293 | 0.038 | 0.162 | 0.220 |  | 0.637 | 0.071 | 0.280 | 0.432 |
|  | 0.6 | 0.354 | 0.043 | 0.337 | 0.358 |  | 0.613 | 0.071 | 0.563 | 0.570 |  | 0.932 | 0.174 | 0.909 | 0.816 |  | 0.609 | 0.076 | 0.378 | 0.499 |  | 0.948 | 0.175 | 0.644 | 0.798 |
|  | 1.0 | 0.761 | 0.108 | 0.716 | 0.691 |  | 0.965 | 0.229 | 0.931 | 0.835 |  | 1.000 | 0.566 | 0.999 | 0.914 |  | 0.980 | 0.233 | 0.821 | 0.907 |  | 1.000 | 0.146 | 0.972 | 0.934 |
| *n*=10 |  |  |  |  |  |  |  |  |  |  |  |  |  |  |  |  |  |  |  |  |  |  |  |  |  |
| 0% | 0.4 | 0.416 | 0.092 | 0.320 | 0.352 |  | 0.690 | 0.168 | 0.543 | 0.554 |  | 0.973 | 0.403 | 0.895 | 0.735 |  | 0.715 | 0.167 | 0.351 | 0.590 |  | 0.984 | 0.401 | 0.629 | 0.899 |
|  | 0.6 | 0.742 | 0.192 | 0.597 | 0.580 |  | 0.955 | 0.363 | 0.858 | 0.722 |  | 1.000 | 0.771 | 0.997 | 0.822 |  | 0.972 | 0.362 | 0.725 | 0.889 |  | 1.000 | 0.752 | 0.937 | 0.945 |
|  | 1.0 | 0.993 | 0.495 | 0.949 | 0.752 |  | 1.000 | 0.813 | 0.998 | 0.835 |  | 1.000 | 0.997 | 1.000 | 0.924 |  | 1.000 | 0.816 | 0.987 | 0.933 |  | 1.000 | 0.992 | 1.000 | 0.935 |
| 20% | 0.4 | 0.334 | 0.075 | 0.320 | 0.305 |  | 0.572 | 0.123 | 0.543 | 0.489 |  | 0.916 | 0.280 | 0.895 | 0.703 |  | 0.573 | 0.124 | 0.351 | 0.480 |  | 0.934 | 0.273 | 0.629 | 0.816 |
|  | 0.6 | 0.635 | 0.148 | 0.597 | 0.509 |  | 0.901 | 0.278 | 0.858 | 0.693 |  | 0.999 | 0.602 | 0.997 | 0.779 |  | 0.921 | 0.270 | 0.725 | 0.826 |  | 1.000 | 0.581 | 0.937 | 0.945 |
|  | 1.0 | 0.967 | 0.386 | 0.949 | 0.727 |  | 0.999 | 0.690 | 0.998 | 0.802 |  | 1.000 | 0.973 | 1.000 | 0.910 |  | 1.000 | 0.691 | 0.987 | 0.923 |  | 1.000 | 0.966 | 1.000 | 0.929 |
| *n*=20 |  |  |  |  |  |  |  |  |  |  |  |  |  |  |  |  |  |  |  |  |  |  |  |  |  |
| 0% | 0.4 | 0.702 | 0.209 | 0.555 | 0.483 |  | 0.940 | 0.383 | 0.827 | 0.617 |  | 1.000 | 0.768 | 0.995 | 0.664 |  | 0.965 | 0.381 | 0.674 | 0.894 |  | 1.000 | 0.758 | 0.919 | 0.973 |
|  | 0.6 | 0.963 | 0.428 | 0.878 | 0.620 |  | 1.000 | 0.721 | 0.991 | 0.653 |  | 1.000 | 0.986 | 1.000 | 0.820 |  | 1.000 | 0.724 | 0.965 | 0.942 |  | 1.000 | 0.982 | 1.000 | 0.952 |
|  | 1.0 | 1.000 | 0.858 | 1.000 | 0.694 |  | 1.000 | 0.993 | 1.000 | 0.837 |  | 1.000 | 1.000 | 1.000 | 0.936 |  | 1.000 | 0.993 | 1.000 | 0.941 |  | 1.000 | 1.000 | 1.000 | 0.944 |
| 20% | 0.4 | 0.587 | 0.167 | 0.555 | 0.427 |  | 0.875 | 0.291 | 0.827 | 0.583 |  | 0.999 | 0.614 | 0.995 | 0.642 |  | 0.899 | 0.293 | 0.674 | 0.811 |  | 1.000 | 0.607 | 0.919 | 0.964 |
|  | 0.6 | 0.913 | 0.338 | 0.878 | 0.601 |  | 0.998 | 0.596 | 0.991 | 0.650 |  | 1.000 | 0.938 | 1.000 | 0.748 |  | 1.000 | 0.192 | 0.965 | 0.940 |  | 1.000 | 0.929 | 1.000 | 0.949 |
|  | 1.0 | 1.000 | 0.758 | 1.000 | 0.664 |  | 1.000 | 0.963 | 1.000 | 0.790 |  | 1.000 | 1.000 | 1.000 | 0.907 |  | 1.000 | 0.966 | 1.000 | 0.933 |  | 1.000 | 0.998 | 1.000 | 0.944 |
| *n*=30 |  |  |  |  |  |  |  |  |  |  |  |  |  |  |  |  |  |  |  |  |  |  |  |  |  |
| 0% | 0.4 | 0.860 | 0.308 | 0.731 | 0.494 |  | 0.991 | 0.560 | 0.941 | 0.534 |  | 1.000 | 0.922 | 1.000 | 0.604 |  | 0.996 | 0.565 | 0.868 | 0.950 |  | 1.000 | 0.912 | 0.989 | 0.977 |
|  | 0.6 | 0.995 | 0.608 | 0.963 | 0.544 |  | 1.000 | 0.892 | 0.999 | 0.579 |  | 1.000 | 0.999 | 1.000 | 0.805 |  | 1.000 | 0.898 | 0.996 | 0.942 |  | 1.000 | 0.998 | 1.000 | 0.964 |
|  | 1.0 | 1.000 | 0.964 | 1.000 | 0.645 |  | 1.000 | 1.000 | 1.000 | 0.833 |  | 1.000 | 1.000 | 1.000 | 0.943 |  | 1.000 | 1.000 | 1.000 | 0.945 |  | 1.000 | 1.000 | 1.000 | 0.958 |
| 20% | 0.4 | 0.782 | 0.249 | 0.731 | 0.458 |  | 0.967 | 0.436 | 0.941 | 0.539 |  | 1.000 | 0.828 | 1.000 | 0.569 |  | 0.982 | 0.442 | 0.868 | 0.929 |  | 1.000 | 0.817 | 0.989 | 0.970 |
|  | 0.6 | 0.982 | 0.498 | 0.963 | 0.539 |  | 1.000 | 0.803 | 0.999 | 0.561 |  | 1.000 | 0.990 | 1.000 | 0.731 |  | 1.000 | 0.802 | 0.996 | 0.939 |  | 1.000 | 0.989 | 1.000 | 0.949 |
|  | 1.0 | 1.000 | 0.920 | 1.000 | 0.599 |  | 1.000 | 0.995 | 1.000 | 0.767 |  | 1.000 | 1.000 | 1.000 | 0.926 |  | 1.000 | 0.995 | 1.000 | 0.933 |  | 1.000 | 0.999 | 1.000 | 0.940 |

M1: Model 1; M2: Model 2; M3: Model 3; M4: Model 4. N/A: Meta-analysis was not available for *n*=1 subject.
