# Supplementary material for: A Comparison of Four Methods for the Analysis of N-of-1 Trials
Source: PLoS One. 2014 Feb 4;9(2):e87752. doi: 10.1371/journal.pone.0087752 (PMC3913644; doi:10.1371/journal.pone.0087752)
Supplement: Table S3 — Bias (PE, %) of 4-cycles N-of-1 trials ( n = 1, 3, 5, 10, 20, 30). (DOC) [file pone.0087752.s003.doc]

**Table S3. Bias (PE, %) of 4-cycles N-of-1 trials (*n*=1, 3, 5, 10, 20, 30).**

| Carryover | Effect | CS1 | | | |  | CS2 | | | |  | CS3 | | | |  | AR1 | | | |  | AR2 | | | |
| --- | --- | --- | --- | --- | --- | --- | --- | --- | --- | --- | --- | --- | --- | --- | --- | --- | --- | --- | --- | --- | --- | --- | --- | --- | --- |
| rate | difference | M1 | M2 | M3 | M4 |  | M1 | M2 | M3 | M4 |  | M1 | M2 | M3 | M4 |  | M1 | M2 | M3 | M4 |  | M1 | M2 | M3 | M4 |
| *n*=1 |  |  |  |  |  |  |  |  |  |  |  |  |  |  |  |  |  |  |  |  |  |  |  |  |  |
| 0% | 0.0 | -0.003 | -0.003 | 0.001 | N/A |  | -0.002 | -0.002 | -0.002 | N/A |  | -0.001 | -0.001 | -0.001 | N/A |  | -0.001 | -0.001 | 0.000 | N/A |  | -0.001 | -0.001 | 0.004 | N/A |
|  | 0.4 | -0.003 (0.7) | -0.003 (0.7) | 0.001 (0.2) | N/A |  | -0.002 (0.5) | -0.002 (0.5) | -0.002 (0.4) | N/A |  | -0.001 (0.3) | -0.001 (0.3) | -0.001 (0.3) | N/A |  | -0.040 (10.1) | -0.040 (10.1) | -0.010 (2.4) | N/A |  | -0.001 (0.2) | -0.001 (0.2) | 0.004 (1.0) | N/A |
|  | 0.6 | -0.003 (0.5) | -0.003 (0.5) | 0.001 (0.1) | N/A |  | -0.002 (0.4) | -0.002 (0.4) | -0.002 (0.3) | N/A |  | -0.001 (0.2) | -0.001 (0.2) | -0.001 (0.2) | N/A |  | -0.060 (10.0) | -0.060 (10.0) | -0.015 (2.4) | N/A |  | -0.001 (0.1) | -0.001 (0.1) | 0.004 (0.6) | N/A |
|  | 1.0 | -0.003 (0.3) | -0.003 (0.3) | 0.001 (0.1) | N/A |  | -0.002 (0.2) | -0.002 (0.2) | -0.002 (0.2) | N/A |  | -0.001 (0.1) | -0.001 (0.1) | -0.001 (0.1) | N/A |  | -0.100 (10.0) | -0.100 (10.0) | -0.025 (2.5) | N/A |  | -0.001 (0.1) | -0.001 (0.1) | 0.004 (0.4) | N/A |
| 20% | 0.0 | -0.002 | -0.002 | 0.001 | N/A |  | -0.001 | -0.001 | -0.002 | N/A |  | -0.001 | -0.001 | -0.001 | N/A |  | -0.001 | -0.001 | 0.000 | N/A |  | 0.000 | 0.000 | 0.004 | N/A |
|  | 0.4 | -0.042 (10.4) | -0.042 (10.4) | -0.009 (2.3) | N/A |  | -0.041 (10.2) | -0.041 (10.2) | -0.012 (2.9) | N/A |  | -0.040 (10.0) | -0.040 (10.0) | -0.011 (2.8) | N/A |  | -0.040 (10.1) | -0.040 (10.1) | -0.010 (2.4) | N/A |  | -0.040 (9.9) | -0.040 (9.9) | -0.006 (1.5) | N/A |
|  | 0.6 | -0.062 (10.3) | -0.062 (10.3) | -0.014 (2.4) | N/A |  | -0.061 (10.1) | -0.061 (10.1) | -0.017 (2.8) | N/A |  | -0.060 (10.0) | -0.060 (10.0) | -0.016 (2.7) | N/A |  | -0.060 (10.0) | -0.060 (10.0) | -0.015 (2.4) | N/A |  | -0.059 (9.9) | -0.059 (9.9) | -0.011 (1.8) | N/A |
|  | 1.0 | -0.101 (10.1) | -0.101 (10.1) | -0.024 (2.4) | N/A |  | -0.100 (10.0) | -0.100 (10.0) | -0.027 (2.7) | N/A |  | -0.100 (10.0) | -0.100 (10.0) | -0.026 (2.6) | N/A |  | -0.100 (10.0) | -0.100 (10.0) | -0.025 (2.5) | N/A |  | -0.099 (9.9) | -0.099 (9.9) | -0.021 (2.1) | N/A |
| *n*=3 |  |  |  |  |  |  |  |  |  |  |  |  |  |  |  |  |  |  |  |  |  |  |  |  |  |
| 0% | 0.0 | -0.001 | 0.001 | -0.008 | 0.002 |  | 0.000 | 0.001 | -0.004 | 0.002 |  | 0.000 | 0.000 | -0.002 | 0.002 |  | -0.001 | 0.000 | -0.004 | 0.001 |  | 0.000 | 0.000 | 0.001 | 0.001 |
|  | 0.4 | -0.001 (0.1) | 0.001 (0.2) | -0.008 (2.1) | 0.063 (15.6) |  | 0.000 (0.1) | 0.001 (0.1) | -0.004 (0.9) | 0.253 (63.2) |  | 0.000 (0.0) | 0.001 (0.1) | -0.002 (0.4) | 0.631 (157.8) |  | -0.001 (0.2) | 0.000 (0.1) | -0.004 (1.0) | 0.115 (28.9) |  | 0.000 (0.0) | 0.000 (0.1) | 0.001 (0.2) | 0.309 (77.3) |
|  | 0.6 | -0.001 (0.1) | 0.001 (0.1) | -0.008 (1.4) | 0.093 (15.5) |  | 0.000 (0.0) | 0.001 (0.1) | -0.004 (0.6) | 0.378 (63.1) |  | 0.000 (0.0) | 0.001 (0.1) | -0.002 (0.3) | 0.946 (157.6) |  | -0.001 (0.1) | 0.000 (0.1) | -0.004 (0.6) | 0.173 (28.8) |  | 0.000 (0.0) | 0.000 (0.1) | 0.001 (0.2) | 0.463 (77.2) |
|  | 1.0 | -0.001 (0.1) | 0.001 (0.1) | -0.008 (0.8) | 0.154 (15.4) |  | 0.000 (0.0) | 0.001 (0.1) | -0.004 (0.4) | 0.630 (63.0) |  | 0.000 (0.0) | 0.001 (0.0) | -0.002 (0.2) | 1.575 (157.5) |  | -0.001 (0.1) | 0.000 (0.0) | -0.004 (0.4) | 0.288 (28.8) |  | 0.000 (0.0) | 0.000 (0.0) | 0.001 (0.1) | 0.771 (77.1) |
| 20% | 0.0 | -0.001 | 0.001 | -0.008 | 0.003 |  | 0.000 | 0.001 | -0.004 | 0.004 |  | 0.000 | 0.000 | -0.002 | 0.004 |  | -0.001 | 0.000 | -0.004 | 0.007 |  | 0.000 | 0.000 | -0.002 | 0.014 |
|  | 0.4 | -0.001 (0.1) | 0.001 (0.2) | -0.008 (2.1) | 0.012 (3.1) |  | -0.041 (10.2) | -0.040 (9.9) | -0.004 (0.9) | 0.175 (43.9) |  | -0.041 (10.1) | -0.040 (9.9) | -0.002 (0.4) | 0.477 (119.4) |  | -0.041 (10.3) | -0.040 (10.1) | -0.004 (1.0) | 0.063 (15.8) |  | -0.04 (10.1) | -0.040 (10.1) | 0.001 (0.2) | 0.231 (57.9) |
|  | 0.6 | -0.061 (10.2) | -0.059 (9.9) | -0.008 (1.4) | 0.016 (2.7) |  | -0.061 (10.1) | -0.060 (9.9) | -0.004 (0.6) | 0.258 (43.1) |  | -0.061 (10.1) | -0.060 (10.0) | -0.002 (0.3) | 0.703 (117.2) |  | -0.061 (10.2) | -0.252 (42.0) | -0.004 (0.6) | 0.090 (15.0) |  | -0.061 (10.1) | -0.060 (10.1) | 0.001 (0.2) | 0.335 (55.9) |
|  | 1.0 | -0.102 (10.2) | -0.099 (9.9) | -0.008 (0.8) | 0.021 (2.1) |  | -0.101 (10.1) | -0.100 (10.0) | -0.004 (0.4) | 0.416 (41.6) |  | -0.101 (10.1) | -0.100 (10.0) | -0.002 (0.2) | 1.124 (112.4) |  | -0.102 (10.2) | -0.101 (10.1) | -0.004 (0.4) | 0.139 (13.9) |  | -0.101 (10.1) | -0.100 (10.0) | 0.001 (0.1) | 0.531 (53.1) |
| *n*=5 |  |  |  |  |  |  |  |  |  |  |  |  |  |  |  |  |  |  |  |  |  |  |  |  |  |
| 0% | 0.0 | -0.003 | -0.001 | -0.006 | -0.006 |  | -0.002 | -0.001 | -0.004 | -0.006 |  | -0.001 | 0.000 | -0.003 | -0.006 |  | -0.001 | 0.003 | -0.005 | -0.004 |  | -0.001 | -0.001 | -0.007 | -0.002 |
|  | 0.4 | -0.003 (0.7) | -0.001 (0.2) | -0.006 (1.5) | 0.055 (13.7) |  | -0.002 (0.5) | -0.001 (0.2) | -0.004 (1.1) | 0.245 (61.2) |  | -0.001 (0.3) | 0.000 (0.1) | -0.003 (0.7) | 0.623 (155.8) |  | -0.001 (0.3) | 0.003 (0.7) | -0.005 (1.3) | 0.113 (28.2) |  | -0.001 (0.4) | -0.001 (0.4) | -0.007 (1.8) | 0.306 (76.5) |
|  | 0.6 | -0.003 (0.5) | -0.001 (0.1) | -0.006 (1.0) | 0.085 (14.2) |  | -0.002 (0.3) | -0.001 (0.1) | -0.004 (0.7) | 0.370 (61.7) |  | -0.001 (0.2) | 0.000 (0.1) | -0.003 (0.5) | 0.938 (156.3) |  | -0.001 (0.2) | 0.003 (0.5) | -0.005 (0.9) | 0.171 (28.6) |  | -0.001 (0.2) | -0.001 (0.2) | -0.007 (1.2) | 0.460 (76.7) |
|  | 1.0 | -0.003 (0.3) | -0.001 (0.1) | -0.006 (0.6) | 0.146 (14.6) |  | -0.002 (0.2) | -0.001 (0.1) | -0.004 (0.4) | 0.621 (62.1) |  | -0.001 (0.1) | 0.000 (0.0) | -0.003 (0.3) | 1.567 (156.7) |  | -0.001 (0.1) | 0.003 (0.3) | -0.005 (0.5) | 0.288 (28.8) |  | -0.001 (0.1) | -0.001 (0.1) | -0.007 (0.7) | 0.768 (76.8) |
| 20% | 0.0 | -0.002 | -0.001 | -0.006 | -0.007 |  | -0.002 | -0.001 | -0.004 | -0.004 |  | -0.001 | 0.000 | -0.003 | -0.002 |  | -0.001 | 0.003 | -0.005 | 0.001 |  | -0.001 | 0.000 | -0.007 | 0.013 |
|  | 0.4 | -0.043 (10.7) | -0.041 (10.3) | -0.006 (1.5) | 0.002 (0.6) |  | -0.042 (10.5) | -0.041 (10.3) | -0.004 (1.1) | 0.166 (41.5) |  | -0.041 (10.4) | -0.041 (10.2) | -0.003 (0.7) | 0.465 (116.2) |  | -0.041 (10.4) | -0.038 (9.4) | -0.005 (1.3) | 0.058 (14.5) |  | -0.042 (10.4) | -0.039 (9.7) | -0.007 (1.8) | 0.229 (57.2) |
|  | 0.6 | -0.063 (10.5) | -0.062 (10.3) | -0.006 (1.0) | 0.006 (1.0) |  | -0.062 (10.4) | -0.061 (10.2) | -0.004 (0.7) | 0.247 (41.2) |  | -0.062 (10.3) | -0.061 (10.2) | -0.003 (0.5) | 0.686 (114.4) |  | -0.062 (10.3) | -0.058 (9.6) | -0.005 (0.9) | 0.085 (14.1) |  | -0.062 (10.3) | -0.060 (10.0) | -0.007 (1.2) | 0.332 (55.4) |
|  | 1.0 | -0.104 (10.4) | -0.102 (10.2) | -0.006 (0.6) | 0.009 (0.9) |  | -0.103 (10.3) | -0.102 (10.2) | -0.004 (0.4) | 0.401 (40.1) |  | -0.102 (10.2) | -0.101 (10.1) | -0.003 (0.3) | 1.098 (109.8) |  | -0.102 (10.2) | -0.098 (9.8) | -0.005 (0.5) | 0.133 (13.3) |  | -0.102 (10.2) | -0.250 (25.0) | -0.007 (0.7) | 0.527 (52.7) |
| *n*=10 |  |  |  |  |  |  |  |  |  |  |  |  |  |  |  |  |  |  |  |  |  |  |  |  |  |
| 0% | 0.0 | -0.001 | 0.000 | 0.002 | -0.002 |  | -0.001 | 0.000 | 0.001 | -0.003 |  | 0.000 | 0.000 | 0.001 | -0.003 |  | -0.001 | 0.000 | 0.000 | -0.001 |  | -0.001 | 0.001 | 0.001 | -0.002 |
|  | 0.4 | -0.001 (0.3) | 0.000 (0.1) | 0.002 (0.5) | 0.061 (15.1) |  | -0.001 (0.2) | 0.000 (0.0) | 0.001 (0.4) | 0.252 (63.0) |  | 0.000 (0.1) | 0.000 (0.0) | 0.001 (0.2) | 0.633 (158.2) |  | -0.001 (0.3) | 0.000 (0.0) | 0.000 (0.1) | 0.115 (28.7) |  | -0.001 (0.1) | 0.001 (0.1) | 0.001 (0.2) | 0.313 (78.2) |
|  | 0.6 | -0.001 (0.2) | 0.000 (0.0) | 0.002 (0.4) | 0.092 (15.3) |  | -0.001 (0.1) | 0.000 (0.0) | 0.001 (0.2) | 0.380 (63.3) |  | 0.000 (0.1) | 0.000 (0.0) | 0.001 (0.1) | 0.951 (158.4) |  | -0.001 (0.2) | 0.000 (0.0) | 0.000 (0.1) | 0.173 (28.8) |  | -0.001 (0.1) | 0.001 (0.1) | 0.001 (0.1) | 0.471 (78.4) |
|  | 1.0 | -0.001 (0.1) | 0.000 (0.0) | 0.002 (0.2) | 0.154 (15.4) |  | -0.001 (0.1) | 0.000 (0.0) | 0.001 (0.1) | 0.635 (63.5) |  | 0.000 (0.0) | 0.000 (0.0) | 0.001 (0.1) | 1.586 (158.6) |  | -0.001 (0.1) | 0.000 (0.0) | 0.000 (0.0) | 0.288 (28.8) |  | -0.001 (0.1) | 0.001 (0.1) | 0.001 (0.1) | 0.786 (78.6) |
| 20% | 0.0 | -0.001 | 0.000 | 0.002 | -0.002 |  | -0.001 | 0.000 | 0.001 | -0.003 |  | 0.000 | 0.000 | 0.001 | -0.002 |  | -0.001 | 0.000 | 0.000 | 0.005 |  | -0.001 | 0.000 | 0.001 | 0.010 |
|  | 0.4 | -0.043 (10.7) | -0.042 (10.5) | 0.002 (0.5) | 0.007 (1.7) |  | -0.042 (10.6) | -0.042 (10.5) | 0.001 (0.4) | 0.168 (42.1) |  | -0.042 (10.5) | -0.042 (10.5) | 0.001 (0.2) | 0.467 (116.7) |  | -0.043 (10.7) | -0.042 (10.6) | 0.000 (0.1) | 0.060 (14.9) |  | -0.042 (10.5) | -0.043( 10.7) | 0.001 (0.2) | 0.229 (57.2) |
|  | 0.6 | -0.064 (10.6) | -0.063 (10.5) | 0.002 (0.4) | 0.010 (1.7) |  | -0.063 (10.5) | -0.063 (10.5) | 0.001 (0.2) | 0.251 (41.8) |  | -0.063 (10.5) | -0.063 (10.5) | 0.001 (0.1) | 0.690 (115.0) |  | -0.063 (10.6) | -0.063 (10.5) | 0.000 (0.1) | 0.085 (14.2) |  | -0.063 (10.5) | -0.064 (10.6) | 0.001 (0.1) | 0.334 (55.6) |
|  | 1.0 | -0.105 (10.5) | -0.105 (10.5) | 0.002 (0.2) | 0.013 (1.3) |  | -0.105 (10.5) | -0.105 (10.5) | 0.001 (0.1) | 0.406 (40.6) |  | -0.104 (10.4) | -0.105 (10.5) | 0.001 (0.1) | 1.105 (110.5) |  | -0.105 (10.5) | -0.105 (10.5) | 0.000 (0.0) | 0.132 (13.2) |  | -0.104 (10.4) | -0.105 (10.5) | 0.001 (0.1) | 0.530 (53.0) |
| *n*=20 |  |  |  |  |  |  |  |  |  |  |  |  |  |  |  |  |  |  |  |  |  |  |  |  |  |
| 0% | 0.0 | -0.002 | 0.000 | -0.002 | -0.003 |  | -0.001 | 0.000 | -0.001 | -0.002 |  | -0.001 | 0.000 | -0.001 | -0.002 |  | -0.002 | -0.001 | -0.002 | -0.001 |  | 0.000 | 0.000 | -0.001 | -0.001 |
|  | 0.4 | -0.002 (0.5) | 0.000 (0.1) | -0.002 (0.5) | 0.058 (14.5) |  | -0.001 (0.3) | 0.000 (0.0) | -0.001 (0.4) | 0.249 (62.2) |  | -0.001 (0.2) | 0.000 (0.0) | -0.001 (0.2) | 0.627 (156.8) |  | -0.002 (0.4) | -0.001 (0.2) | -0.002 (0.5) | 0.113 (28.3) |  | 0.000 (0.1) | 0.000 (0.1) | -0.001 (0.3) | 0.305 (76.3) |
|  | 0.6 | -0.002 (0.3) | 0.000 (0.0) | -0.002 (0.3) | 0.088 (14.7) |  | -0.001 (0.2) | 0.000 (0.0) | -0.001 (0.2) | 0.374 (62.4) |  | -0.001 (0.1) | 0.000 (0.0) | -0.001 (0.1) | 0.942 (157.0) |  | -0.002 (0.3) | -0.001 (0.1) | -0.002(0.3) | 0.171 (28.5) |  | 0.000 (0.1) | 0.000 (0.1) | -0.001 (0.2) | 0.458 (76.4) |
|  | 1.0 | -0.002 (0.2) | 0.000 (0.0) | -0.002 (0.2) | 0.149 (14.9) |  | -0.001 (0.1) | 0.000 (0.0) | -0.001 (0.1) | 0.626 (62.6) |  | -0.001 (0.1) | 0.000 (0.0) | -0.001 (0.1) | 1.571 (157.1) |  | -0.002 (0.2) | -0.001 (0.1) | -0.002 (0.2) | 0.286 (28.6) |  | 0.000 (0.1) | 0.000 (0.0) | -0.001 (0.1) | 0.764 (76.4) |
| 20% | 0.0 | -0.002 | 0.000 | -0.002 | -0.003 |  | -0.001 | 0.000 | -0.001 | -0.003 |  | -0.001 | 0.000 | -0.001 | -0.003 |  | -0.002 | -0.001 | -0.002 | 0.003 |  | 0.000 | 0.000 | -0.001 | 0.011 |
|  | 0.4 | -0.043 (10.9) | -0.042 (10.5) | -0.002 (0.5) | 0.005 (1.2) |  | -0.043 (10.7) | -0.042 (10.5) | -0.001 (0.4) | 0.166 (41.6) |  | -0.042 (10.5) | -0.042 (10.5) | -0.001 (0.2) | 0.465 (116.2) |  | -0.043 (10.7) | -0.043 (10.7) | -0.002 (0.5) | 0.058 (14.4) |  | -0.042 (10.4) | -0.043 (10.7) | -0.001 (0.3) | 0.224 (56.1) |
|  | 0.6 | -0.064 (10.7) | -0.063 (10.5) | -0.002 (0.3) | 0.007 (1.2) |  | -0.063 (10.6) | -0.063 (10.5) | -0.001 (0.2) | 0.248 (41.3) |  | -0.063 (10.5) | -0.063 (10.5) | -0.001 (0.1) | 0.687 (114.6) |  | -0.063 (10.6) | -0.374 (62.4) | -0.002(0.3) | 0.083 (13.9) |  | -0.062 (10.4) | -0.064 (10.6) | -0.001 (0.2) | 0.326 (54.4) |
|  | 1.0 | -0.105 (10.5) | -0.105 (10.5) | -0.002 (0.2) | 0.010 (1.0) |  | -0.105 (10.5) | -0.105 (10.5) | -0.001 (0.1) | 0.402 (40.2) |  | -0.104 (10.4) | -0.105 (10.5) | -0.001 (0.1) | 1.102 (110.2) |  | -0.105 (10.5) | -0.106 (10.6) | -0.002 (0.2) | 0.129 (12.9) |  | -0.104 (10.4) | -0.105 (10.5) | -0.001 (0.1) | 0.517 (51.7) |
| *n*=30 |  |  |  |  |  |  |  |  |  |  |  |  |  |  |  |  |  |  |  |  |  |  |  |  |  |
| 0% | 0.0 | -0.002 | 0.000 | -0.003 | -0.002 |  | -0.002 | 0.000 | -0.002 | -0.003 |  | -0.001 | 0.000 | -0.001 | -0.003 |  | -0.002 | 0.000 | -0.002 | -0.001 |  | -0.001 | 0.000 | -0.001 | -0.002 |
|  | 0.4 | -0.002 (0.6) | 0.000 (0.1) | -0.003 (0.8) | 0.059 (14.8) |  | -0.002 (0.4) | 0.000 (0.1) | -0.002 (0.6) | 0.249 (62.4) |  | -0.001 (0.2) | 0.000 (0.0) | -0.001 (0.3) | 0.628 (157.1) |  | -0.002 (0.4) | 0.000 (0.1) | -0.002 (0.6) | 0.113 (28.3) |  | -0.001 (0.2) | 0.000 (0.1) | -0.001 (0.3) | 0.306 (76.4) |
|  | 0.6 | -0.002 (0.4) | 0.000 (0.1) | -0.003 (0.6) | 0.090 (14.9) |  | -0.002 (0.3) | 0.000 (0.0) | -0.002 (0.4) | 0.375 (62.6) |  | -0.001 (0.2) | 0.000 (0.0) | -0.001 (0.2) | 0.944 (157.3) |  | -0.002 (0.3) | 0.000 (0.0) | -0.002 (0.4) | 0.171 (28.4) |  | -0.001 (0.1) | 0.000 (0.1) | -0.001 (0.2) | 0.459 (76.6) |
|  | 1.0 | -0.002 (0.2) | 0.000 (0.0) | -0.003 (0.3) | 0.151 (15.1) |  | -0.002 (0.2) | 0.000 (0.0) | -0.002 (0.2) | 0.627 (62.7) |  | -0.001 (0.1) | 0.000 (0.0) | -0.001 (0.1) | 1.575 (157.5) |  | -0.002 (0.2) | 0.000 (0.0) | -0.002 (0.2) | 0.285 (28.5) |  | -0.001 (0.1) | 0.000 (0.0) | -0.001 (0.1) | 0.767 (76.7) |
| 20% | 0.0 | -0.002 | 0.000 | -0.003 | -0.002 |  | -0.002 | 0.000 | -0.002 | -0.003 |  | -0.001 | 0.000 | -0.001 | -0.003 |  | -0.002 | 0.000 | -0.002 | 0.004 |  | -0.001 | 0.000 | -0.001 | 0.010 |
|  | 0.4 | -0.043 (10.9) | -0.042 (10.5) | -0.003 (0.8) | 0.006 (1.6) |  | -0.043 (10.7) | -0.042 (10.5) | -0.002 (0.6) | 0.168 (42.0) |  | -0.042 (10.5) | -0.042 (10.4) | -0.001 (0.3) | 0.466 (116.6) |  | -0.043 (10.6) | -0.041 (10.3) | -0.002 (0.6) | 0.058 (14.6) |  | -0.042 (10.5) | -0.041 (10.3) | -0.001 (0.3) | 0.226 (56.4) |
|  | 0.6 | -0.064 (10.7) | -0.063 (10.4) | -0.003 (0.6) | 0.009 (1.6) |  | -0.063 (10.5) | -0.063 (10.4) | -0.002 (0.4) | 0.252 (41.9) |  | -0.063 (10.4) | -0.063 (10.4) | -0.001 (0.2) | 0.690 (115.0) |  | -0.007 (1.1) | -0.062 (10.4) | -0.002 (0.4) | 0.084 (14) |  | -0.062 (10.4) | -0.062 (10.3) | -0.001 (0.2) | 0.329 (54.8) |
|  | 1.0 | -0.105 (10.5) | -0.104 (10.4) | -0.003 (0.3) | 0.012 (1.2) |  | -0.104 (10.4) | -0.104 (10.4) | -0.002 (0.2) | 0.405 (40.5) |  | -0.104 (10.4) | -0.104 (10.4) | -0.001 (0.1) | 1.105 (110.5) |  | -0.104 (10.4) | -0.104 (10.4) | -0.002 (0.2) | 0.130 (13.0) |  | -0.103 (10.3) | -0.104 (10.4) | -0.001 (0.1) | 0.522 (52.2) |

M1: Model 1; M2: Model 2; M3: Model 3; M4: Model 4. N/A: Meta-analysis was not available for *n*=1 subject.
