# Supplementary material for: A Comparison of Four Methods for the Analysis of N-of-1 Trials
Source: PLoS One. 2014 Feb 4;9(2):e87752. doi: 10.1371/journal.pone.0087752 (PMC3913644; doi:10.1371/journal.pone.0087752)
Supplement: Table S4 — MSE of 4-cycles N-of-1 trials ( n = 1, 3, 5, 10, 20, 30). (DOC) [file pone.0087752.s004.doc]

**Table S4. MSE of 4-cycles N-of-1 trials (*n*=1, 3, 5, 10, 20, 30).**

| Carryover | Effect | CS1 | | | |  | CS2 | | | |  | CS3 | | | |  | AR1 | | | |  | AR2 | | | |
| --- | --- | --- | --- | --- | --- | --- | --- | --- | --- | --- | --- | --- | --- | --- | --- | --- | --- | --- | --- | --- | --- | --- | --- | --- | --- |
| rate | difference | M1 | M2 | M3 | M4 |  | M1 | M2 | M3 | M4 |  | M1 | M2 | M3 | M4 |  | M1 | M2 | M3 | M4 |  | M1 | M2 | M3 | M4 |
| *n*=1 |  |  |  |  |  |  |  |  |  |  |  |  |  |  |  |  |  |  |  |  |  |  |  |  |  |
| 0% | 0.0 | 0.503 | 0.503 | 0.899 | N/A |  | 0.252 | 0.252 | 0.450 | N/A |  | 0.101 | 0.101 | 0.180 | N/A |  | 0.204 | 0.204 | 0.503 | N/A |  | 0.081 | 0.081 | 0.259 | N/A |
|  | 0.4 | 0.503 | 0.503 | 0.899 | N/A |  | 0.252 | 0.252 | 0.450 | N/A |  | 0.101 | 0.101 | 0.180 | N/A |  | 0.208 | 0.208 | 0.504 | N/A |  | 0.081 | 0.081 | 0.259 | N/A |
|  | 0.6 | 0.503 | 0.503 | 0.899 | N/A |  | 0.252 | 0.252 | 0.450 | N/A |  | 0.101 | 0.101 | 0.180 | N/A |  | 0.211 | 0.211 | 0.505 | N/A |  | 0.081 | 0.081 | 0.259 | N/A |
|  | 1.0 | 0.503 | 0.503 | 0.899 | N/A |  | 0.252 | 0.252 | 0.450 | N/A |  | 0.101 | 0.101 | 0.180 | N/A |  | 0.221 | 0.221 | 0.509 | N/A |  | 0.081 | 0.081 | 0.259 | N/A |
| 20% | 0.0 | 0.510 | 0.510 | 0.899 | N/A |  | 0.259 | 0.259 | 0.450 | N/A |  | 0.109 | 0.109 | 0.180 | N/A |  | 0.204 | 0.204 | 0.503 | N/A |  | 0.093 | 0.093 | 0.259 | N/A |
|  | 0.4 | 0.514 | 0.514 | 0.900 | N/A |  | 0.263 | 0.263 | 0.451 | N/A |  | 0.113 | 0.113 | 0.181 | N/A |  | 0.208 | 0.208 | 0.504 | N/A |  | 0.097 | 0.097 | 0.260 | N/A |
|  | 0.6 | 0.518 | 0.518 | 0.901 | N/A |  | 0.267 | 0.267 | 0.453 | N/A |  | 0.117 | 0.117 | 0.182 | N/A |  | 0.211 | 0.211 | 0.505 | N/A |  | 0.100 | 0.100 | 0.261 | N/A |
|  | 1.0 | 0.527 | 0.527 | 0.905 | N/A |  | 0.276 | 0.276 | 0.456 | N/A |  | 0.126 | 0.126 | 0.186 | N/A |  | 0.221 | 0.221 | 0.509 | N/A |  | 0.110 | 0.110 | 0.264 | N/A |
| *n*=3 |  |  |  |  |  |  |  |  |  |  |  |  |  |  |  |  |  |  |  |  |  |  |  |  |  |
| 0% | 0.0 | 0.178 | 0.670 | 0.326 | 0.264 |  | 0.090 | 0.335 | 0.168 | 0.264 |  | 0.036 | 0.134 | 0.067 | 0.264 |  | 0.068 | 0.337 | 0.195 | 0.125 |  | 0.028 | 0.133 | 0.105 | 0.094 |
|  | 0.4 | 0.178 | 0.670 | 0.326 | 0.280 |  | 0.090 | 0.335 | 0.168 | 0.352 |  | 0.036 | 0.134 | 0.067 | 0.717 |  | 0.068 | 0.337 | 0.195 | 0.152 |  | 0.028 | 0.133 | 0.105 | 0.223 |
|  | 0.6 | 0.178 | 0.670 | 0.326 | 0.298 |  | 0.090 | 0.335 | 0.168 | 0.456 |  | 0.036 | 0.134 | 0.067 | 1.275 |  | 0.068 | 0.337 | 0.195 | 0.186 |  | 0.028 | 0.133 | 0.105 | 0.382 |
|  | 1.0 | 0.178 | 0.670 | 0.326 | 0.353 |  | 0.090 | 0.335 | 0.168 | 0.788 |  | 0.036 | 0.134 | 0.067 | 3.053 |  | 0.068 | 0.337 | 0.195 | 0.294 |  | 0.028 | 0.133 | 0.105 | 0.891 |
| 20% | 0.0 | 0.178 | 0.670 | 0.326 | 0.257 |  | 0.090 | 0.335 | 0.168 | 0.253 |  | 0.036 | 0.134 | 0.067 | 0.243 |  | 0.068 | 0.337 | 0.194 | 0.121 |  | 0.029 | 0.133 | 0.092 | 0.091 |
|  | 0.4 | 0.178 | 0.670 | 0.326 | 0.265 |  | 0.092 | 0.337 | 0.168 | 0.297 |  | 0.038 | 0.136 | 0.067 | 0.501 |  | 0.071 | 0.339 | 0.195 | 0.139 |  | 0.031 | 0.135 | 0.092 | 0.173 |
|  | 0.6 | 0.182 | 0.675 | 0.326 | 0.275 |  | 0.094 | 0.340 | 0.168 | 0.350 |  | 0.041 | 0.139 | 0.067 | 0.805 |  | 0.073 | 0.107 | 0.195 | 0.158 |  | 0.033 | 0.137 | 0.092 | 0.263 |
|  | 1.0 | 0.189 | 0.684 | 0.326 | 0.303 |  | 0.102 | 0.349 | 0.168 | 0.508 |  | 0.048 | 0.148 | 0.067 | 1.690 |  | 0.080 | 0.350 | 0.195 | 0.213 |  | 0.040 | 0.146 | 0.092 | 0.524 |
| *n*=5 |  |  |  |  |  |  |  |  |  |  |  |  |  |  |  |  |  |  |  |  |  |  |  |  |  |
| 0% | 0.0 | 0.092 | 0.400 | 0.145 | 0.143 |  | 0.047 | 0.200 | 0.074 | 0.145 |  | 0.019 | 0.080 | 0.030 | 0.146 |  | 0.035 | 0.194 | 0.086 | 0.068 |  | 0.016 | 0.081 | 0.040 | 0.052 |
|  | 0.4 | 0.092 | 0.400 | 0.145 | 0.151 |  | 0.047 | 0.200 | 0.074 | 0.215 |  | 0.019 | 0.080 | 0.030 | 0.561 |  | 0.035 | 0.194 | 0.086 | 0.090 |  | 0.016 | 0.081 | 0.040 | 0.166 |
|  | 0.6 | 0.092 | 0.400 | 0.145 | 0.163 |  | 0.047 | 0.200 | 0.074 | 0.307 |  | 0.019 | 0.080 | 0.030 | 1.089 |  | 0.035 | 0.194 | 0.086 | 0.118 |  | 0.016 | 0.081 | 0.040 | 0.308 |
|  | 1.0 | 0.092 | 0.400 | 0.145 | 0.202 |  | 0.047 | 0.200 | 0.074 | 0.603 |  | 0.019 | 0.080 | 0.030 | 2.783 |  | 0.035 | 0.194 | 0.086 | 0.209 |  | 0.016 | 0.081 | 0.040 | 0.764 |
| 20% | 0.0 | 0.093 | 0.400 | 0.145 | 0.142 |  | 0.047 | 0.200 | 0.074 | 0.142 |  | 0.019 | 0.080 | 0.030 | 0.138 |  | 0.036 | 0.194 | 0.086 | 0.068 |  | 0.016 | 0.080 | 0.040 | 0.052 |
|  | 0.4 | 0.095 | 0.402 | 0.145 | 0.146 |  | 0.049 | 0.202 | 0.074 | 0.176 |  | 0.021 | 0.082 | 0.030 | 0.371 |  | 0.038 | 0.196 | 0.086 | 0.080 |  | 0.018 | 0.081 | 0.040 | 0.122 |
|  | 0.6 | 0.097 | 0.405 | 0.145 | 0.152 |  | 0.052 | 0.205 | 0.074 | 0.220 |  | 0.023 | 0.084 | 0.030 | 0.646 |  | 0.040 | 0.199 | 0.086 | 0.093 |  | 0.020 | 0.084 | 0.040 | 0.199 |
|  | 1.0 | 0.105 | 0.413 | 0.145 | 0.170 |  | 0.059 | 0.212 | 0.074 | 0.349 |  | 0.031 | 0.092 | 0.030 | 1.438 |  | 0.047 | 0.206 | 0.086 | 0.132 |  | 0.027 | 0.093 | 0.040 | 0.421 |
| *n*=10 |  |  |  |  |  |  |  |  |  |  |  |  |  |  |  |  |  |  |  |  |  |  |  |  |  |
| 0% | 0.0 | 0.051 | 0.193 | 0.077 | 0.077 |  | 0.025 | 0.096 | 0.038 | 0.076 |  | 0.010 | 0.039 | 0.015 | 0.075 |  | 0.020 | 0.096 | 0.041 | 0.037 |  | 0.008 | 0.039 | 0.019 | 0.026 |
|  | 0.4 | 0.051 | 0.193 | 0.077 | 0.083 |  | 0.025 | 0.096 | 0.038 | 0.144 |  | 0.010 | 0.039 | 0.015 | 0.489 |  | 0.020 | 0.096 | 0.041 | 0.055 |  | 0.008 | 0.039 | 0.019 | 0.135 |
|  | 0.6 | 0.051 | 0.193 | 0.077 | 0.092 |  | 0.025 | 0.096 | 0.038 | 0.232 |  | 0.010 | 0.039 | 0.015 | 1.010 |  | 0.020 | 0.096 | 0.041 | 0.077 |  | 0.008 | 0.039 | 0.019 | 0.271 |
|  | 1.0 | 0.051 | 0.193 | 0.077 | 0.118 |  | 0.025 | 0.096 | 0.038 | 0.513 |  | 0.010 | 0.039 | 0.015 | 2.679 |  | 0.020 | 0.096 | 0.041 | 0.147 |  | 0.008 | 0.039 | 0.019 | 0.707 |
| 20% | 0.0 | 0.051 | 0.193 | 0.077 | 0.074 |  | 0.025 | 0.096 | 0.038 | 0.072 |  | 0.010 | 0.039 | 0.015 | 0.068 |  | 0.020 | 0.096 | 0.041 | 0.036 |  | 0.008 | 0.039 | 0.019 | 0.025 |
|  | 0.4 | 0.052 | 0.195 | 0.077 | 0.076 |  | 0.027 | 0.098 | 0.038 | 0.104 |  | 0.012 | 0.041 | 0.015 | 0.293 |  | 0.022 | 0.098 | 0.041 | 0.043 |  | 0.010 | 0.041 | 0.019 | 0.086 |
|  | 0.6 | 0.055 | 0.197 | 0.077 | 0.079 |  | 0.029 | 0.101 | 0.038 | 0.142 |  | 0.014 | 0.043 | 0.015 | 0.562 |  | 0.024 | 0.101 | 0.041 | 0.051 |  | 0.012 | 0.044 | 0.019 | 0.154 |
|  | 1.0 | 0.062 | 0.205 | 0.077 | 0.087 |  | 0.036 | 0.109 | 0.038 | 0.259 |  | 0.021 | 0.051 | 0.015 | 1.339 |  | 0.031 | 0.109 | 0.041 | 0.074 |  | 0.019 | 0.052 | 0.019 | 0.351 |
| *n*=20 |  |  |  |  |  |  |  |  |  |  |  |  |  |  |  |  |  |  |  |  |  |  |  |  |  |
| 0% | 0.0 | 0.025 | 0.099 | 0.036 | 0.037 |  | 0.013 | 0.050 | 0.018 | 0.037 |  | 0.005 | 0.020 | 0.007 | 0.037 |  | 0.010 | 0.049 | 0.020 | 0.018 |  | 0.004 | 0.019 | 0.010 | 0.013 |
|  | 0.4 | 0.025 | 0.099 | 0.036 | 0.042 |  | 0.013 | 0.050 | 0.018 | 0.102 |  | 0.005 | 0.020 | 0.007 | 0.437 |  | 0.010 | 0.049 | 0.020 | 0.033 |  | 0.004 | 0.019 | 0.010 | 0.111 |
|  | 0.6 | 0.025 | 0.099 | 0.036 | 0.048 |  | 0.013 | 0.050 | 0.018 | 0.183 |  | 0.005 | 0.020 | 0.007 | 0.939 |  | 0.010 | 0.049 | 0.020 | 0.052 |  | 0.004 | 0.019 | 0.010 | 0.234 |
|  | 1.0 | 0.025 | 0.099 | 0.036 | 0.067 |  | 0.013 | 0.050 | 0.018 | 0.445 |  | 0.005 | 0.020 | 0.007 | 2.548 |  | 0.010 | 0.049 | 0.020 | 0.112 |  | 0.004 | 0.019 | 0.010 | 0.628 |
| 20% | 0.0 | 0.025 | 0.099 | 0.036 | 0.037 |  | 0.013 | 0.050 | 0.018 | 0.036 |  | 0.005 | 0.020 | 0.007 | 0.035 |  | 0.010 | 0.049 | 0.020 | 0.018 |  | 0.004 | 0.019 | 0.010 | 0.013 |
|  | 0.4 | 0.027 | 0.101 | 0.036 | 0.038 |  | 0.014 | 0.052 | 0.018 | 0.065 |  | 0.007 | 0.022 | 0.007 | 0.254 |  | 0.011 | 0.051 | 0.020 | 0.023 |  | 0.006 | 0.022 | 0.010 | 0.067 |
|  | 0.6 | 0.029 | 0.104 | 0.036 | 0.039 |  | 0.017 | 0.054 | 0.018 | 0.101 |  | 0.009 | 0.024 | 0.007 | 0.515 |  | 0.014 | 0.198 | 0.020 | 0.029 |  | 0.008 | 0.024 | 0.010 | 0.128 |
|  | 1.0 | 0.036 | 0.111 | 0.036 | 0.043 |  | 0.024 | 0.061 | 0.018 | 0.209 |  | 0.016 | 0.032 | 0.007 | 1.273 |  | 0.021 | 0.061 | 0.020 | 0.045 |  | 0.015 | 0.031 | 0.010 | 0.302 |
| *n*=30 |  |  |  |  |  |  |  |  |  |  |  |  |  |  |  |  |  |  |  |  |  |  |  |  |  |
| 0% | 0.0 | 0.017 | 0.067 | 0.025 | 0.026 |  | 0.008 | 0.034 | 0.013 | 0.026 |  | 0.003 | 0.013 | 0.005 | 0.026 |  | 0.007 | 0.034 | 0.013 | 0.012 |  | 0.003 | 0.014 | 0.006 | 0.009 |
|  | 0.4 | 0.017 | 0.067 | 0.025 | 0.030 |  | 0.008 | 0.034 | 0.013 | 0.090 |  | 0.003 | 0.013 | 0.005 | 0.425 |  | 0.007 | 0.034 | 0.013 | 0.027 |  | 0.003 | 0.014 | 0.006 | 0.105 |
|  | 0.6 | 0.017 | 0.067 | 0.025 | 0.036 |  | 0.008 | 0.034 | 0.013 | 0.171 |  | 0.003 | 0.013 | 0.005 | 0.927 |  | 0.007 | 0.034 | 0.013 | 0.045 |  | 0.003 | 0.014 | 0.006 | 0.227 |
|  | 1.0 | 0.017 | 0.067 | 0.025 | 0.055 |  | 0.008 | 0.034 | 0.013 | 0.432 |  | 0.003 | 0.013 | 0.005 | 2.536 |  | 0.007 | 0.034 | 0.013 | 0.103 |  | 0.003 | 0.014 | 0.006 | 0.617 |
| 20% | 0.0 | 0.017 | 0.067 | 0.025 | 0.025 |  | 0.008 | 0.034 | 0.013 | 0.025 |  | 0.003 | 0.013 | 0.005 | 0.024 |  | 0.007 | 0.034 | 0.013 | 0.012 |  | 0.003 | 0.014 | 0.006 | 0.009 |
|  | 0.4 | 0.019 | 0.069 | 0.025 | 0.026 |  | 0.010 | 0.035 | 0.013 | 0.055 |  | 0.005 | 0.015 | 0.005 | 0.244 |  | 0.008 | 0.036 | 0.013 | 0.017 |  | 0.004 | 0.015 | 0.006 | 0.062 |
|  | 0.6 | 0.021 | 0.071 | 0.025 | 0.027 |  | 0.012 | 0.038 | 0.013 | 0.091 |  | 0.007 | 0.017 | 0.005 | 0.507 |  | 0.001 | 0.038 | 0.013 | 0.022 |  | 0.007 | 0.018 | 0.006 | 0.123 |
|  | 1.0 | 0.028 | 0.078 | 0.025 | 0.030 |  | 0.019 | 0.045 | 0.013 | 0.198 |  | 0.014 | 0.025 | 0.005 | 1.264 |  | 0.018 | 0.045 | 0.013 | 0.036 |  | 0.013 | 0.025 | 0.006 | 0.297 |

M1: Model 1; M2: Model 2; M3: Model 3; M4: Model 4. N/A: Meta-analysis was not available for *n*=1 subject.
